# Structural distinctions between NAD^+^ riboswitch domains 1 and 2 determine differential folding and ligand binding

**DOI:** 10.1093/nar/gkaa1029

**Published:** 2020-11-10

**Authors:** Hao Chen, Michaela Egger, Xiaochen Xu, Laurin Flemmich, Olga Krasheninina, Aiai Sun, Ronald Micura, Aiming Ren

**Affiliations:** Life Sciences Institute, Zhejiang University, Hangzhou, Zhejiang 310058, China; Institute of Organic Chemistry, Center for Molecular Biosciences Innsbruck, University of Innsbruck, Innsbruck, 6020, Austria; Life Sciences Institute, Zhejiang University, Hangzhou, Zhejiang 310058, China; Institute of Organic Chemistry, Center for Molecular Biosciences Innsbruck, University of Innsbruck, Innsbruck, 6020, Austria; Institute of Organic Chemistry, Center for Molecular Biosciences Innsbruck, University of Innsbruck, Innsbruck, 6020, Austria; School of Chemistry and Materials Science, Hangzhou Institute for Advanced Study, University of Chinese Academy of Sciences, 1 Sub-lane Xiangshan, Hangzhou 310024, China; Institute of Organic Chemistry, Center for Molecular Biosciences Innsbruck, University of Innsbruck, Innsbruck, 6020, Austria; Life Sciences Institute, Zhejiang University, Hangzhou, Zhejiang 310058, China

## Abstract

Riboswitches are important gene regulatory elements frequently encountered in bacterial mRNAs. The recently discovered *nadA* riboswitch contains two similar, tandemly arrayed aptamer domains, with the first domain possessing high affinity for nicotinamide adenine dinucleotide (NAD^+^). The second domain which comprises the ribosomal binding site in a putative regulatory helix, however, has withdrawn from detection of ligand-induced structural modulation thus far, and therefore, the identity of the cognate ligand and the regulation mechanism have remained unclear. Here, we report crystal structures of both riboswitch domains, each bound to NAD^+^. Furthermore, we demonstrate that ligand binding to domain 2 requires significantly higher concentrations of NAD^+^ (or ADP retaining analogs) compared to domain 1. Using a fluorescence spectroscopic approach, we further shed light on the structural features which are responsible for the different ligand affinities, and describe the Mg^2+^-dependent, distinct folding and pre-organization of their binding pockets. Finally, we speculate about possible scenarios for *nadA* RNA gene regulation as a putative two-concentration sensor module for a time-controlled signal that is primed and stalled by the gene regulation machinery at low ligand concentrations (domain 1), and finally triggers repression of translation as soon as high ligand concentrations are reached in the cell (domain 2).

## INTRODUCTION

Riboswitches are gene regulatory elements that are frequently found in the 5′-untranslated regions of bacterial mRNAs (1–6). They are usually organized by an aptamer domain that binds the cognate ligand with high specificity, and upon binding, a folding path of the nascent mRNA is entered that is distinct from the path in absence of the ligand (7,8). Hence, the 3′-aptamer–adjoining RNA sequence—the expression platform—exhibits mutually exclusive secondary structures which transmit the signal ON or OFF for gene expression. In most cases, riboswitches act at either the transcriptional level (terminator/anti-terminator stem) or translational level (repressor/anti-repressor stem), and they regulate the expression of downstream genes which encode for proteins involved in the production and transport of the small molecule that binds to the riboswitch by this kind of a feed-back mechanism. It is noteworthy that some riboswitches are organized with tandem components, for instance, the glycine riboswitch is composed of two homologous ligand-binding domains that each bind glycine. The two aptamers act together to regulate the expression of glycine metabolic and transport genes (9–13). Other tandem riboswitches form arrangements to approximate the function of specific two-input Boolean logic gates, as for instance has been recently revealed for a phosphoribosyl pyrophosphate (PRPP) and guanine sensing riboswitch system (14).

The first description of riboswitches dates back to the early years of the twenty-first century (15–18), and ever since more than forty riboswitches for different ligand types have been identified in all three domains of life (19). The most abundant riboswitches regulate common enzyme cofactors such as adenosylcobalamin, thiamin pyrophosphate (TPP), flavin mononucleotide, *S*-adenosylmethionine or tetrahydrofolate. Only very recently, the *nadA* RNA motif has been assigned to function as a riboswitch which regulates the expression of genes involved in biosynthesis of the enzyme cofactor nicotinamide adenine dinucleotide (NAD^+^) (20). Although the cofactor is ubiquitous, this riboswitch appears to be rather rare and has the special feature of a tandem aptamer architecture. Early *in vitro* assays implied that NAD^+^ is bound with an apparent 1:1 stoichiometry, and with *K*_d_ values that are in the 100 μM range (20). The binding was attributed entirely due to recognition of the 5′ adenosine diphosphate (ADP) moiety of the cofactor by the first aptamer domain (20), consistent with a recent crystal structure of domain 1 bound to NAD^+^ and analogs (21). Interestingly, for the second putative aptamer that harbours the ribosomal binding site (Shine Dalgarno) in the regulator stem, no ligand-induced structural modulation was observed (20), and the regulation mechanism of this riboswitch and the precise function of the individual domains has remained unclear.

Here, we report crystal structures of the first, as well as the second domain of the NAD^+^ riboswitch, both individually bound to NAD^+^. We furthermore demonstrate that ligand binding to the second domain requires significantly higher concentrations of the ligand. We also shed light on the structural matters that are responsible for the different ligand affinities of the two domains as well as determine the Mg^2+^-dependent, distinct folding and pre-organization of their binding pockets. Finally, we reflect on possible scenarios for the gene regulation mechanism of this intriguing riboswitch.

## MATERIALS AND METHODS

### Preparation of RNA for crystallization

To facilitate crystallization of *nadA* RNA constructs, GNRA tetra-loop motifs and/or the U1A-protein recognition loop were introduced as apical variable loop of stem P1b of domain 1 and domain 2, respectively (Supplementary Table S1). The sequence of the riboswitch, followed by the sequence of the HDV ribozyme was cloned into pUT7 plasmids bearing a T7 RNA polymerase promoter, which was amplified in *Escherichia coli* cells and linearized by endonuclease *Hind* III delivering the template for transcription (22). *In vitro* transcription was carried out at 37°C using T7 RNA polymerase, followed by purification with denatured polyacrylamide gel electrophoresis (PAGE). The product RNA was visualized using ultraviolet light at a wavelength of 254 nm, excised and soaked in 0.5 × Tris-acetate-EDTA (TAE) buffer at 4°C. The leach solution was precipitated with iso-propanol and washed by 80% ethanol. The lyophilized RNA was dissolved in diethyl pyrocarbonate treated, double-distilled water for further experiments.

### U1A protein production

The U1A protein (2–98 aa, Y31H/Q36R) with an N-terminal His_10_-SUMO-tag and an ubiquitin-like protease (ULP1) cleavage site was expressed in *E. coli* (BL21-Codon Plus^®^) at 18°C overnight. The harvested cell pellets were suspended in buffer A (25 mM Tris, pH 8.0, 500 mM NaCl, 5 mM 2-mercaptoethanol) and lysed with a French press. After high-speed centrifugation, the supernatant was loaded on a Ni^2+^ column. The His_10_-SUMO-tag recombinant U1A protein was eluted using buffer A supplemented with 500 mM imidazole and then cleaved with ULP1, which was followed by a second Ni^2+^ column to remove the cleaved His_10_-SUMO-tag. The U1A protein was further purified with ion-exchange (HiTrap SP HP column, GE Healthcare) and gel-filtration (HiLoad Superdex 75 16/600 column, GE Healthcare) chromatography methods, and finally concentrated to 20 mg/ml for further experiments. The selenomethionine (Se-Met) derivative of the U1A protein was expressed in cells grown in M9 medium supplemented with Se-Met and other amino acids. The purification process of Se-Met U1A was the same as for the native protein.

### Ligands

The ligands NAD^+^, NADH, adenosine 5'-diphosphate ADP and adenosine 5'-triphosphate (ATP) were purchased from Yuanye Bio-Technology Co., Ltd (Shanghai). Nicotine mononucleotide (NMN) was purchased from Bide Pharmatech Ltd (Shanghai).

### Crystallization, structure determination and refinement

A final concentration of 0.4 mM NAD^+^ riboswitch RNA in the buffer containing 50 mM HEPES, pH 7.0, 50 mM NaCl, 5 mM MgCl_2_ was annealed at 65°C for 5 min and then incubated on ice for half an hour. NAD^+^ was added to the RNA with a ratio of RNA to NAD^+^ 1:15 and kept on ice for one more hour. For the constructs containing the U1A-recognition loop, U1A protein was added with the ratio of RNA to U1A protein 1:1.2. Crystallization experiments were carried out by mixing 0.16 µl of the RNA-ligand complex with reservoir solution at an equimolar ratio using sitting drop vapour diffusion at 16°C. Well-diffracting crystals of domain 1 construct 17delU1A in complex with NAD^+^ grew from the buffer solutions containing 0.2 M Mg(OAc)_2_, 0.1 M NaAsO_2_, pH 6.5, 30% MPD after 3 days, while the crystals of the domain 1 construct 18GAAA in complex with NAD^+^, ADP and ATP, grew from buffer solutions containing 0.1 M MES pH 6.5, 1.6 M MgSO_4_ after 1 or 2 days. The crystals of domain 2 construct 832GAAA-NAD^+^ grew from buffer solutions containing 0.2 M NaCl, 0.1 M CHES pH 9.5, 50% PEG400 after two weeks. All the crystals were flash-frozen with liquid nitrogen before data collection.

X-ray diffraction data was collected at Shanghai Synchrotron Radiation Facility (SSRF) and processed with HKL3000 (HKL Research). The phase problem of 17delU1A-NAD^+^ was solved based on the anomalous diffraction signal collected from the co-crystallized Se-Met modified U1A protein by the single-wavelength anomalous diffraction (SAD) method using the program Autosol in the Phenix software suite (23). The model was built and refined using the Coot program (24) with Phenix software (23). The structures of 832GAAA-NAD^+^ and 18GAAA with different ligands were solved by molecular replacement (MR) using the Phaser program in the CCP4 software suite with the structure of 17delU1A as the initial model (25).

For Mn^2+^ soaking experiments, the crystals were transferred into the crystallization solution supplemented with 500 mM MnCl_2_ at 4°C for 1 h for 18GAAA-NAD^+^ or with 25 mM MnCl_2_ at 4°C for 1.5 h for 832GAAA-NAD^+^.

All X-ray data collection and crystallographic refinement statistics are listed in Supplementary Table S2.

### Isothermal titration calorimetry

All isothermal titration calorimetry (ITC) experiments during this study were performed on a Microcal PEAQ-ITC instrumentation at the National Center for Protein Science·Shanghai (NCPSS). The RNA samples were dialyzed at 4°C overnight against a buffer containing 50 mM HEPES pH 7.0, 50 mM NaCl, 10 mM MgCl_2_. RNA was refolded by annealing at 65°C for 5 min and followed by incubating on ice for 30 min after dilution to a concentration of 0.1 mM. All ligands were dissolved in the dialysis buffer and diluted to a concentration of 2 mM before titration into the RNA in the sample well (200 μl). The titration experiments were performed at 25°C and initiated with an injection of 0.4 μl, followed by 18 serial injections of 2 μl of the ligand each, with a 120 s interval between each injection and a reference power of 5 μcal s^−1^.

The titration data were integrated and analyzed using MicroCal PEAQ-ITC analysis software. The apparent dissociation constant (*K*_d_) was calculated based on the ‘one set of sites’ binding model. All the binding constants and thermodynamic values are listed in Supplementary Table S3.

### Gel-filtration chromatography

A final concentration of 0.3 mM RNA in 50 mM HEPES, pH 7.0, 50 mM NaCl, 5 mM MgCl_2_ was annealed at 65°C for 5 min and followed by cooling on ice for 1 h. In the NAD^+^-bound form of NAD^+^ riboswitch formation, NAD^+^ was added to the final concentration of 6 mM and then incubated on ice for 1 h. All the samples were loaded on the Superose 10/300 GL 6 Increase column (GE Healthcare) that was pre-equilibrated with the buffer (50 mM HEPES, pH 7.0, 50 mM NaCl, 5 mM MgCl_2_). Data was processed in Microsoft Excel and Origin software.

### Steady-state fluorescence spectroscopy

All steady-state fluorescence spectroscopic experiments were measured on a Cary Eclipse spectrometer (Varian, Australia) equipped with a peltier block, a magnetic stirring device and a RX2000 stopped-flow apparatus (Applied Photophysics Ltd., UK). The data obtained were processed with OriginPro 2018 software (OriginLab, USA).

### Binding affinities

Ap-modified RNA samples were prepared in 0.5 μM concentration in a total volume of 1 ml of buffer (50 mM KMOPS pH 7.5, 100 mM KCl, 50 mM MgCl_2_). The samples were heated to 90°C for 2 min, allowed to cool to room temperature, transferred to quartz cuvettes equipped with a small stir bar and held at 20°C in the peltier controlled sample holder. Then, ligands were manually pipetted in a way not to exceed a total volume increase of 3%. The solution was stirred during each titration step and allowed to equilibrate for at least 15 min before data collection. Spectra were recorded from 330 to 500 nm using the following instrumental parameters: excitation wavelength, 308 nm; increments, 1 nm; scan rate, 120 nm/min; slit widths, 10 nm. The apparent binding constants *K*_d_ were determined by following the increase in fluorescence after each titration step via integration of the area between 330 and 500 nm. Changes in fluorescence (*F* − *F*_0_) were normalized to the maximum fluorescence measured at the maximum concentration of ligand. The measurement for each titration step was repeated at least three times and the mean of the normalized fluorescence intensity and the corresponding error bars for each value were plotted against the ligand concentration. Data were fit using a two-parametric (*K*_d_ and δ) quadratic equation implying 1:1 stoichiometry, according to reference (26). The final Kd value is determined from fitting of data obtained from three independent titration experiments. The standard deviations corresponding to each value of the normalized fluorescence intensity were calculated using an equation:

$$\begin{eqnarray*}&&{\rm{Error\ }}\left( {\frac{{F - {F_0}}}{{{F_f} - {F_0}}}} \right) \\ &&\quad = \frac{{\sqrt {{{\left( {SF} \right)}^2} + {{\left( {\frac{{F - {F_f}}}{{{F_f} - {F_0}}} \cdot S{F_0}} \right)}^2} + {{\left( {\frac{{F - {F_0}}}{{{F_f} - {F_0}}} \cdot S{F_f}} \right)}^2}} }}{{{F_f} - {F_0}}}\ \end{eqnarray*}$$

where SF corresponds to the standard error of the mean (SEM) of fluorescence intensity for each titration step, *SF*_0_ and *SF*_f_ correspond to the SEM of initial and final fluorescence intensities, respectively.

## RESULTS AND DISCUSSION

### Constructs and crystallization of the individual domains of the NAD^+^ riboswitch

Our X-ray structural studies were focused on both individual domains of the NAD^+^ riboswitch. We screened a large number of in vitro transcribed RNA constructs from different species and got two independent structures of domain 1 of the NAD^+^ riboswitch in complex with NAD^+^ and one structure of domain 2 in complex with NAD^+^. The RNA sequences used for structure determination are listed in Supplementary Table S1. The non-conserved sequence portion adjoining to stem P1b in domain 1 was dispensable for NAD^+^ binding as was the non-conserved region adjoining to stem P1b in domain 2. Therefore, they were replaced with GAAA tetra-loops, and in case of domain 1, additionally, with the U1A protein-binding loop in one of the constructs (Supplementary Figure S1B and C).

In co-crystallization with the U1A protein, the crystal structure of the NAD^+^ riboswitch domain 1 (from *Acidobacterium capsulatum ATCC 51196*; in the following named 17delU1A-D1/NAD^+^) was obtained at a resolution of 2.80 Å. The space group was *P*6122, in which each asymmetric unit contained one molecule. The phase problem of 17delU1A-D1/NAD^+^ was solved by the SAD method using the anomalous signal of selenium in selenomethionine (Se-Met)-derivatized U1A protein. The second structure with the GAAA tetra-loop replacement in domain 1 from an environmental sequence of the *nadA* RNA motif (RF03013 Rfam database, https://rfam.xfam.org; named 18GAAA-D1/NAD^+^ in the following) was solved at a resolution of 2.40 Å in space group *I*222. The phase problem of 18GAAA-D1/NAD^+^ was mastered by MR using the trimmed RNA structure of 17delU1A-D1/NAD^+^ as the initial model. In addition, we solved the crystal structures of 18GAAA-D1 in complex with adenosine 5′-diphosphate (ADP) and adenosine 5′-triphosphate (ATP) at a resolution of 2.60 Å and 2.80 Å, respectively.

For the structural studies on domain 2 of the NAD^+^ riboswitch, we used the sequence information from *Acidobateria*ceae bacterium KBS83 (Supplementary Figure S1C). Crystals of this RNA bound with NAD^+^ diffracted to 2.10 Å. The structure was named as 832GAAA-D2/NAD^+^, the space group of 832GAAA-D2/NAD^+^ was *P*3_2_21, in which each asymmetric unit contain one molecule (Supplementary Table S2). The phase problem of the 832GAAA-D1/NAD^+^ structure was solved by MR using the trimmed RNA structure of 17delU1A-D1/NAD^+^ as the initial model.

### Tertiary fold of NAD^+^ riboswitch domain 1 in complex with NAD^+^

**Figure 1.**
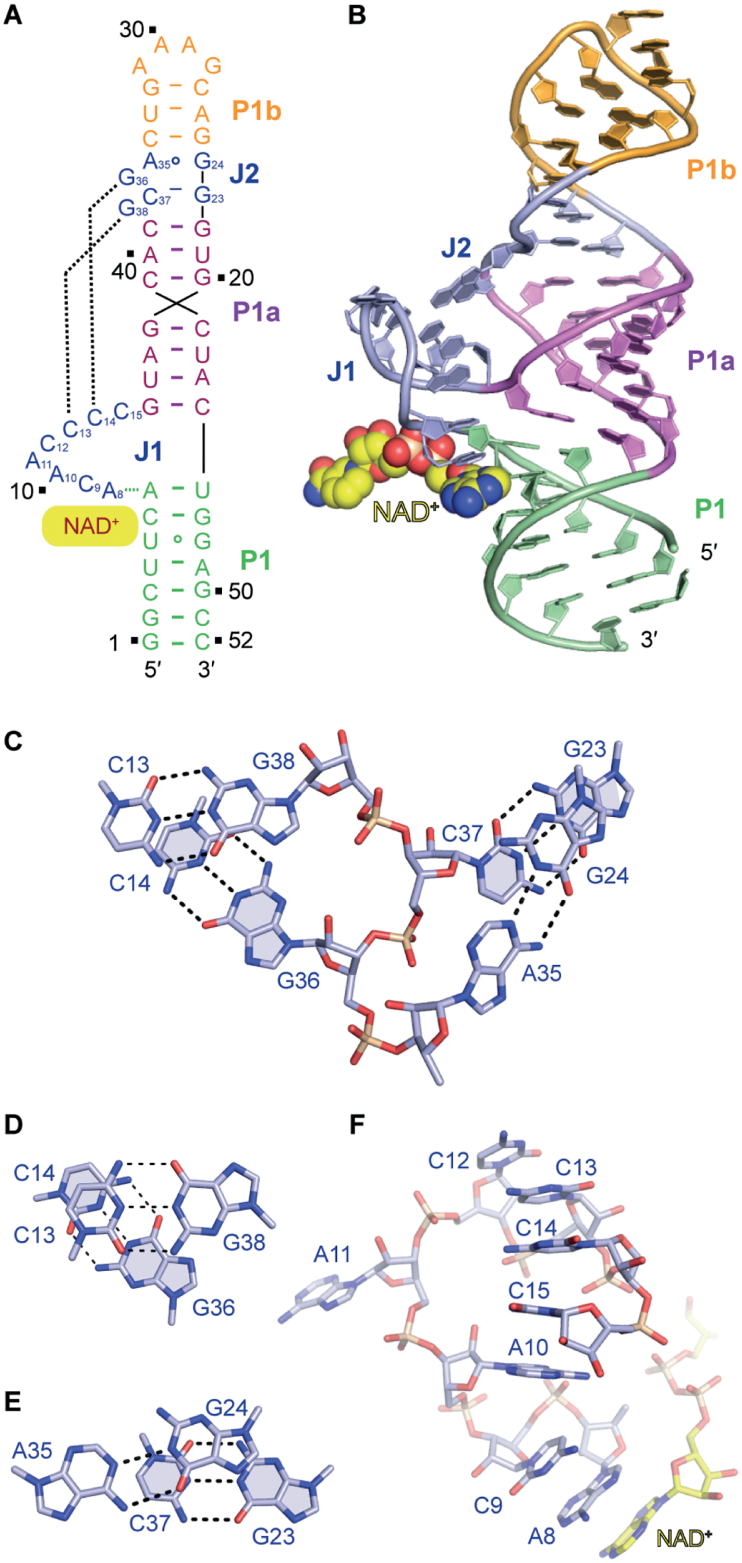
Secondary and tertiary structure of domain 1 of the ligand-bound NAD^+^ riboswitch. (**A**) Secondary structure scheme of the folding topology based on the crystal structure. (**B**) Cartoon representation of the tertiary structure of NAD^+^ riboswitch domain 1. (**C**) Summary of base pairing interactions within and between junctions J1 and J2. (**D**) Close-up view of the J1/J2 long-distance interaction: Watson–Crick base pairs formed by G36 and G38 (J2) with C13 and C14 (J1). (**E**) Close-up view of internal interactions in junction J2: A35 forms a non-canonical base pair with G24, and C37 forms a Watson–Crick base pair with G23. (**F**) Except A11, all the nucleotides in junction J1 stack continuously, including the nucleobase of the ligand NAD^+^.

The tertiary fold of 18GAAA-D1/NAD^+^ that is shown schematically in Figure 1A and in cartoon representation in Figure 1B, conforms to the consensus secondary structure model of domain 1 of the *nadA* motif from phylogenetic analysis (Supplementary Figure S1B). The RNA adopts a pseudoknot scaffold in ‘Y-shape’, in which stems P1b, P1a and P1 form a co-axial long helix, with integrated base pairs from the internal bubble J2 (Figure 1A and B; Supplementary Figure S2A). The junctional loop J1 (residing between P1 and P1a) zippers up and forms a long-distance interaction with J2 (residing between stems P1a and P1b) (Figure 1C–F and Supplementary Figure S2B). NAD^+^ is bound at the intersection of stem P1 and the junction loop J1, employing a type-I A minor base triple (27,28).

All the residues in J1 except A11 stack continuously through their bases above the adenine base of ligand NAD^+^ (Figure 1C–F and Supplementary Figure S2B). Importantly, C13 and C14 from J1 hydrogen bond with G38 and G36 from J2, respectively, forming two regular Watson–Crick base pairs (Figure 1C–F and Supplementary Figure S2B). Furthermore, 4-NH_2_ of C15 forms a hydrogen bond with 2′-OH of A10. At the same time, 6-NH_2_ of A10 forms additional two hydrogen bonds with the non-bridging phosphate oxygen of A8 and the non-bridging phosphate oxygen of G16, respectively (Supplementary Figure S2C). G16 staples on A7 and forms a hydrogen bond with 2′-OH of A7 using the non-bridging phosphate oxygen, thereby constituting continuous stacking between stem P1 and P1a (Supplementary Figure S2C and D). C9 is positioned in the same plane as the terminal base pair G16-C45 of stem P1a and forms two hydrogen bonds with 4′-O of G16 and 2′-OH of A7, which helps to stabilize the formation of the long helix (Supplementary Figure S2C and D). 6-NH_2_ of A8 interacts with the minor groove of the terminal base pair A7-U46 of stem P1 and hence forms the sides of the NAD^+^ binding pocket (Supplementary Figure S2D and E). Four of the six residues in junction J2, namely G23, G24, A35 and C37 interact intensively with each other, forming one non-canonical base pair G24•A35 and one canonical Watson–Crick base pair G23-C37. These base pairs intercalate between stem P1a and P1b (Figure 1A, C and E) and become an integral part of the long helix. All these junction interactions including the long-distance pairing (G38-C13 and G36-C14) stabilize the overall fold, and simultaneously, position A8 of J1 as a key recognition element for NAD^+^ in the RNA binding pocket. Furthermore, we underline that A10, A11 in J1 and G36, C37, G38 in J2 adopt *2*′*-endo* sugar pucker conformation (Figure 1C and F).

The second crystal structure of domain 1 of the NAD^+^ riboswitch-ligand complex (17delU1A-D1/NAD^+^) that we solved independently at slightly lower resolution is illustrated in Supplementary Figure S3A and B. The RNAs 18GAAA-D1 and 17delU1A-D1 are distinct in their stem sequences (P1, P1a and P1b), but with respect to their junctional elements J1 and J2, eleven out of 13 bases are identical (these nucleotides are labeled in red in Supplementary Figure S1C; Supplementary Table S1). Alignment of the tertiary structure of 18GAAA-D1/NAD^+^ and 17delU1A-D1/NAD^+^ shows high compatibility (Supplementary Figure S3C), which complies with the phylogenetic sequence analysis of the NAD^+^ riboswitch. Therefore, it is not surprising that 17delU1A-D1/NAD^+^ folds into a similar pseudoknot scaffold as 18GAAA-D1/NAD^+^, and C12 and C13 from J1 form the characteristic long-distance base-paring interaction with G43 and G45 from J2 (Supplementary Figure S3A). Moreover, the conserved residues of the junctional regions arrange in the same architecture in both crystal structures (Supplementary Figure S3D).

Furthermore, we point out that both complexes, 18GAAA-D1/NAD^+^ and 17delU1A-D1/NAD^+^, form homogenous dimers with their symmetry-related molecules in the crystal (Supplementary Figures S4 and 5). The dimerization interface and the involved interactions are, however, clearly distinct (Supplementary Figures S4 and 5). As shown in Supplementary Figure S4, the junction J1 of 18GAAA-D1/NAD^+^ provides points of contact (Supplementary Figure S4), with the sugar edge of C15 from one molecule hydrogen bonding to NH_2_ of A11′ from the symmetry-related molecule (Supplementary Figure S4C). Additionally, an octahedrally coordinated cation (Mg^2+^) mediates between A10/A11 of one molecule and C15′/G16′/U17′ of the other (Supplementary Figure S4D). In contrast to 18GAAA-D1/NAD^+^, the 17delU1A-D1/NAD^+^ dimer involves both junctional regions J1 and J2 in contacts between the individual units (Supplementary Figure S5). The C11′ from the symmetric molecule forms a Hoogsteen interaction with G45 (Supplementary Figure S5C) and hence generates a base triple C11′•G45-C12 that forms an extended stacking interface (with C11•G45′-C12′) between the two molecules in the dimer (Supplementary Figure S5A and B). To analyze whether domain 1 of the NAD^+^ riboswitch forms dimers in solution, we applied gel-filtration chromatography for RNA 18GAAA-D1 (without/with NAD^+^) and RNA 17delU1A-D1 (without/with NAD^+^). Clearly, under the conditions used, both RNAs existed as monomers in solution without any indication for dimer formation (Supplementary Figure S6). Therefore, the intermolecular RNA interactions observed in the crystal seem to be rather weak. This is also consistent with the different crystal packing observed for the RNA in the two structures.

### Binding pocket interactions of domain 1 with NAD^+^ and related compounds

**Figure 2.**
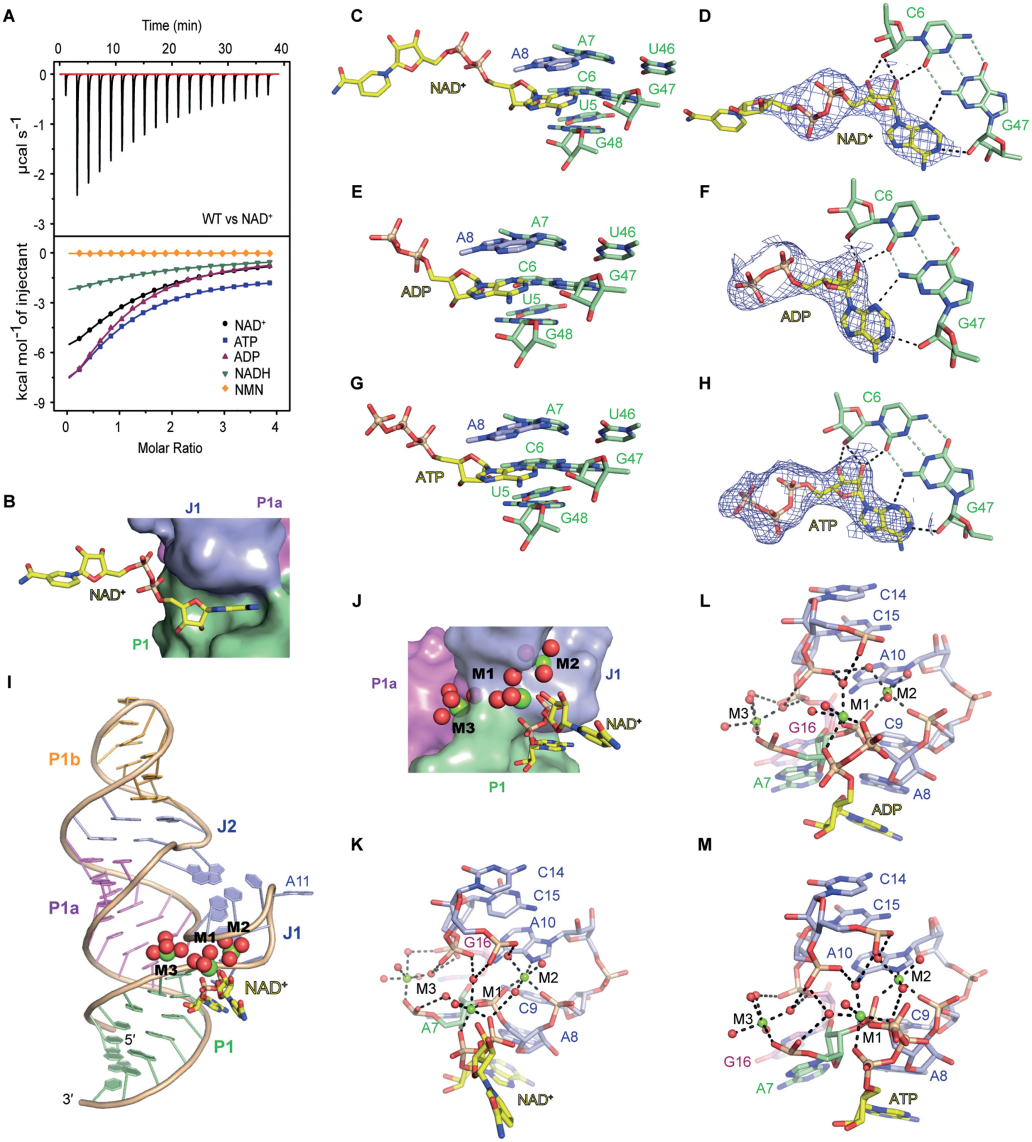
Structural details of ligand interactions of the NAD^+^ riboswitch domain 1. (**A**) Thermogram of exemplary ITC experiment of NAD^+^ riboswitch domain 1 binding to NAD^+^ (top) and overlay of integrated fitted heat plots of NAD^+^, ATP, ADP, NADH and NMN (bottom); for arithmetic mean of *K*_D_ value and thermodynamic parameters see Supplementary Table S3. (**B**) The adenine moiety of NAD^+^ (shown in sticks representation) is buried into a cavity of the RNA binding pocket (shown in surface representation), while the NMN moiety of NAD^+^ points outwards. (**C**–**H**) The adenine moieties of ligands NAD^+^, ADP and ATP are each stacked between base pair G48•U5 and base triple A8•A7-U46 (C, E and G); the adenine together with the ribose moieties of the ligands form intensive hydrogen-bond interaction with the minor groove of the C6-G47 base pair; the composite omit maps (contoured at 1.0 σ level) of the ligands are shown (D, F and H). (**I** and **J**) Three octahedrally coordinated Mg^2+^ cations M1, M2 and M3 are observed in the NAD^+^ binding pocket. (**K**) Close-up view of the coordination of three Mg^2+^ cations to RNA and ligand NAD^+^: M1 forms inner-sphere coordinations with the two phosphate groups of the ligand, and with the phosphate oxygen of A8 (pA8); additionally, M1 forms outer-sphere coordinations with pC14, pC15 and pA7. M2 forms inner-sphere coordinations with N7 of A10, pA8 and pC9; additionally, M2 forms out-sphere coordination with pC14 and one phosphate of NAD^+^. M3 forms inner-sphere coordination with the phosphate oxygen of A7 (pA7) (terminus of stem P1), and outer-sphere coordinations with G16 (terminus of stem P1a) and C15 (J1). (**L** and **M**) The three Mg^2+^ cations M1, M2 and M3 are also observed for NAD^+^ riboswitch domain 1 bound to ADP or ATP; the coordination patterns of the divalent metal ions are the same as for the NAD^+^-bound structure (see panel K).

Early in-line probing experiments indicated that beside NAD^+^, structurally related compounds also bind, in particular those that retain the ADP substructure. To this end, we performed ITC (Figure 2A; Supplementary Figure S7 and Table S2) and found that domain 1 of the NAD^+^ riboswitch binds to its dedicated ligand NAD^+^ with an affinity *K*_d_ of 127 μM. Slightly higher binding affinities were determined for ADP (*K*_d_ = 95.3 μM) and ATP (*K*_d_ = 90.2 μM) while NADH (*K*_d_ = 305 μM) gave a 3-fold decrease in binding affinity compared to NAD^+^. NMN does not bind to domain 1 of the NAD^+^ riboswitch (Figure 2A). These results are consistent with the binding mode revealed by our crystal structures. The surface representation of the binding pocket shows that it is the ADP moiety of NAD^+^ that interacts intensively, with the nucleobase deeply buried into a cavity and the pyrophosphate nestling up the RNA surface, while the NMN moiety of NAD^+^ points outwards without any obvious RNA interactions (Figure 2B). In more detail, the adenine base and the ribose of NAD^+^ form five hydrogen bonds with the minor groove edge of G47-C6 and hence generate a base triple (Figure 2C and D), which is stacked between the base triple A8•A7-U46 (Supplementary Figure S2E) and the non-canonical base pair of U5•G48 (Figure 2C and D). The composite omit map (contoured at 1.0 σ level) of NAD^+^ is shown in Figure 2D.

In addition, we solved the structures of 18GAAA-D1 bound to ADP and to ATP, respectively (Figure 2E–H and Supplementary Figure S8). All three complexes 18GAAA-D1/NAD^+^, 18GAAA-D1/ADP and 18GAAA-D1/ATP adopt very similar architectures, with the superposition of NAD^+^- and ADP-bound RNAs generating an RMSD value of 0.171 Å (Supplementary Figure S8B) and the superposition of NAD^+^- and ATP-bound RNAs generating an RMSD value of 0.322 Å (Supplementary Figure S8D) for all atoms. The interaction pattern between the ADP moiety of the ligands with nucleosides of the 18GAAA-D1 binding pocket is the same for all three complexes (Figure 2E–H).

Three Mg^2+^ cations labeled M1, M2 and M3 were identified in the domain 1 binding pocket of the NAD^+^ riboswitch based on 2*Fo-Fc* and *Fo-Fc* maps guided by the coordination geometries (Figure 2I–J), which were further validated by the observation of anomalous signals collected from Mn^2+^-soaked crystals (Supplementary Figure S9A). The three Mg^2+^ cations are coordinated by water molecules, the ligand NAD^+^ itself and seven nucleotides (A7-A8-C9-A10 and C14-C15-G16) aligning the binding pocket (Figure 2K). Six nucleotides (A7-A8-C9, C14-C15-G16) form multiple coordinations with these hydrated Mg^2+^ ions using their phosphates. Further, the pyrophosphate of NAD^+^ forms a bidentate coordination to one of the Mg^2+^ ions, and one nucleotide (A10) forms inner-sphere coordination with its nucleobase (Figure 2K). More precisely, M1 displays three inner-sphere coordinations to pA8 and to two of the non-bridging phosphate oxygens of the NAD^+^ pyrophosphate moiety, while the remaining coordination sites are water-mediated (outer-sphere) interactions with pA7, pC14 and pC15 (Figure 2K and Supplementary Figure S9B and C). M2 is inner-sphere coordinated with pC9, pA8 and N7 of A10, and additionally is outer-sphere coordinated with the NAD^+^ pyrophosphate and pC14. M3 is inner-sphere coordinated to pA7 and outer-sphere coordinated to pC15 and pG16 (Figure 2K and Supplementary Figure S9C). Except A7 (the terminus of stem P1) and G16 (the terminus of stem P1a), all other nucleotides are residing in junction J1 (Figures 1A and 2K; Supplementary Figure S9B and C). The three Mg^2+^ cations therefore determine the folding of J1, and hence, the NAD^+^ binding pocket, and one of them (M1) is key for mediating the interaction of the negatively charged pyrophosphate of ligand and selected phosphates of the aptamer, thereby creating a crucial recognition feature. Of additional note, the three Mg^2+^ cations were also observed in the binding pocket of ADP- and ATP-bound structures of domain 1 of the NAD^+^ riboswitch (Figure 2L and M).

### Tertiary fold and binding pocket interactions of NAD^+^ riboswitch domain 2 bound to NAD^+^

**Figure 3.**
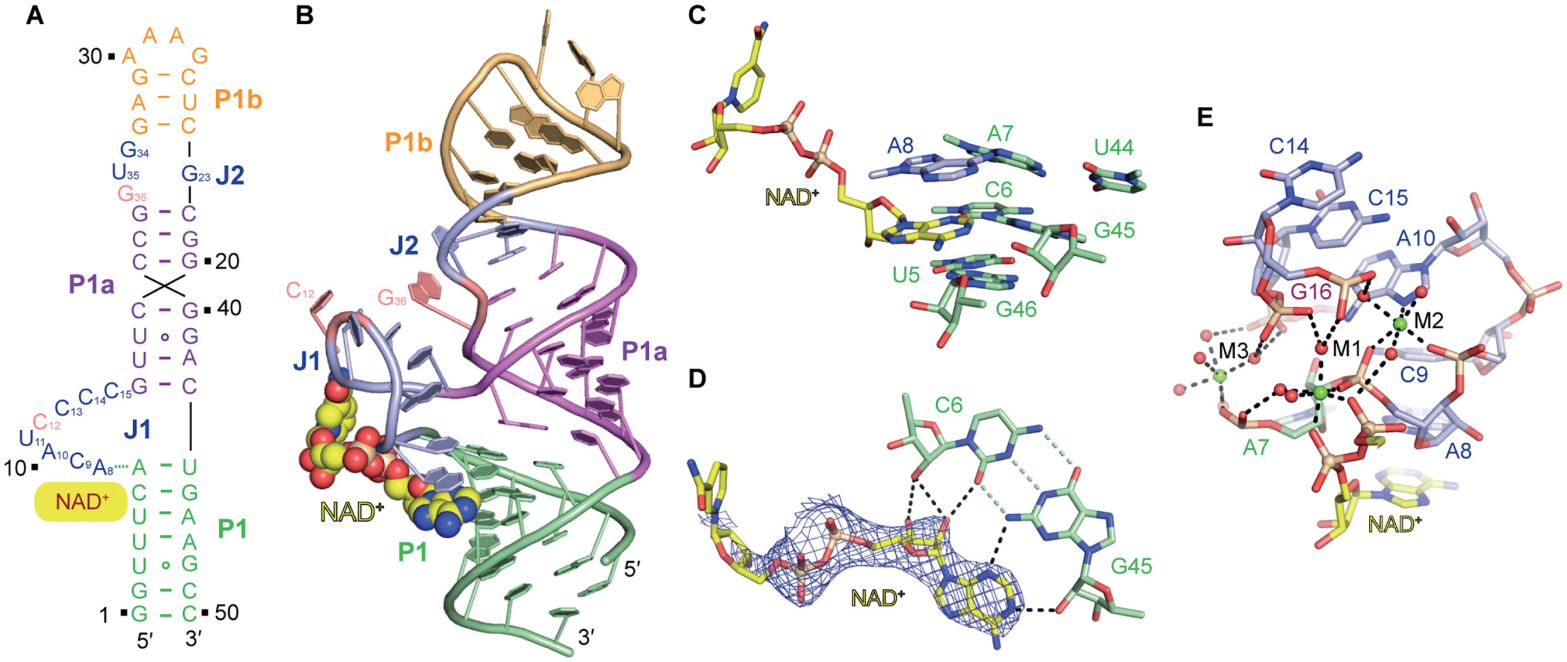
Secondary and tertiary structure of domain 2 of the ligand-bound NAD^+^ riboswitch and details of the binding pocket. (**A**) Secondary structure scheme of the folding topology based on the crystal structure. (**B**) Cartoon representation of the tertiary structure of NAD^+^ riboswitch domain 2. (**C**) Close-up view of the interaction of NAD^+^ ligand with surrounded nucleotides in the ligand binding pocket of domain 2. (**D**) The adenine together with the ribose moiety of NAD^+^ form intensive hydrogen-bond interaction with the minor groove of the C6-G45 base pair; the composite omit map (contoured at 1.0 σ level) of the ligand NAD^+^ is shown. (**E**) Three Mg^2+^ cations are present in the ligand binding pocket of the NAD^+^ riboswitch domain 2 and their coordination pattern is similar to that observed for domain 1.

Though attempts to crystallize the full-length NAD^+^ riboswitch in the presence of NAD^+^ were unsuccessful, we did succeed in generating crystals of domain 2 alone in complex with NAD^+^ (832GAAA-D2/NAD^+^), diffracting at a resolution of 2.1 Å. The structure is shown schematically in Figure 3A and in cartoon representation in Figure 3B. It was unexpected that the NAD^+^ riboswitch domain 2—like domain 1—adopts a ‘Y-shape’ overall topology in complex with NAD^+^ without forming the characteristic long-distance J1-J2 pairing interaction that we see in domain 1 (Figures 1A-B and 3A-B). Also, the ligand NAD^+^ is bound similarly to domain 1, positioned at the intersection of the north terminus of stem P1 and the junction J1 (Figures 1A-B and 3A-B). All stacking and hydrogen bonding interactions are retained (Figure 3C and D; Supplementary Figure S10) and the three octahedrally coordinated Mg^2+^ ions are also present with a similar coordination pattern to NAD^+^ and the nucleotides in the binding pocket of 832GAAA-D2. The identities of the divalent ions were independently confirmed by the anomalous signals observed from Mn^2+^-soaked crystals (Supplementary Figure S10A).

A structural overlay of domain 1 (18GAAA-D1/NAD^+^) and domain 2 (832GAAA-D2/NAD^+^) of the NAD^+^ riboswitch reveals the same alignment of stem P1, J1 and the bound ADP-moiety of the ligand NAD^+^. The long double helical arrangement of stem P1a and P1b, however, shows clear distinctions originating from the altered nucleoside composition of J2 in 832GAAA-D2 that comprises only four instead of the original 6 nt (Supplementary Figure S11A–D). G23 forms a Watson–Crick base pair with C37 in J2 of 18GAAA-D1 (Figure 1A, C and E; Supplementary Figure S2E), however, the corresponding nucleotides G23 and U35 in 832GAAA-D2 do not pair, but form a non-planar base triple interaction with G34 (Figure 3A and Supplementary Figure S11C–E). It is notable that G34 from 832GAAA-D2, whose corresponding nucleotide is G36 in 18GAAA-D1, does not exhibit the long-distance interaction with C14 in J1, but points inwards and interacts with G23-G34-U35 instead (Supplementary Figure S11C and D). The place of G24-A35 in 18GAAA-D1 is taken by the terminal base pair G33-C24 of stem P1b in 832GAAA-D2 (Supplementary Figure S11C), which induces a significant reduction of the base pair width from 20.8 Å (G24-A35 in 18GAAA-D1) to 17.9 Å (G33-C24 in 832GAAA-D2) (Supplementary Figure S11F and G). Furthermore, G36 in 832GAAA-D2, whose corresponding nucleotide is G38 in 18GAAA-D1, also lacks the long-distance pairing to J1 (Figures 1A and 3A; Supplementary Figure S11E) but forms a Watson–Crick base pair with C12′ from a symmetric molecule in the crystal, resulting in symmetry-related dimers of 832GAAA-D2 (Supplementary Figure S12). The NAD^+^ binding pocket present in each molecule of the dimer are on opposite sides and do not interact with each other (Supplementary Figure S12C). Gel-filtration chromatography demonstrated that 832GAAA-D2 exists as monomer in the absence and presence of NAD^+^ in solution (Supplementary Figure S6C).

### Mutational analysis of key nucleotides in domain 1 using ITC for readout

**Figure 4.**
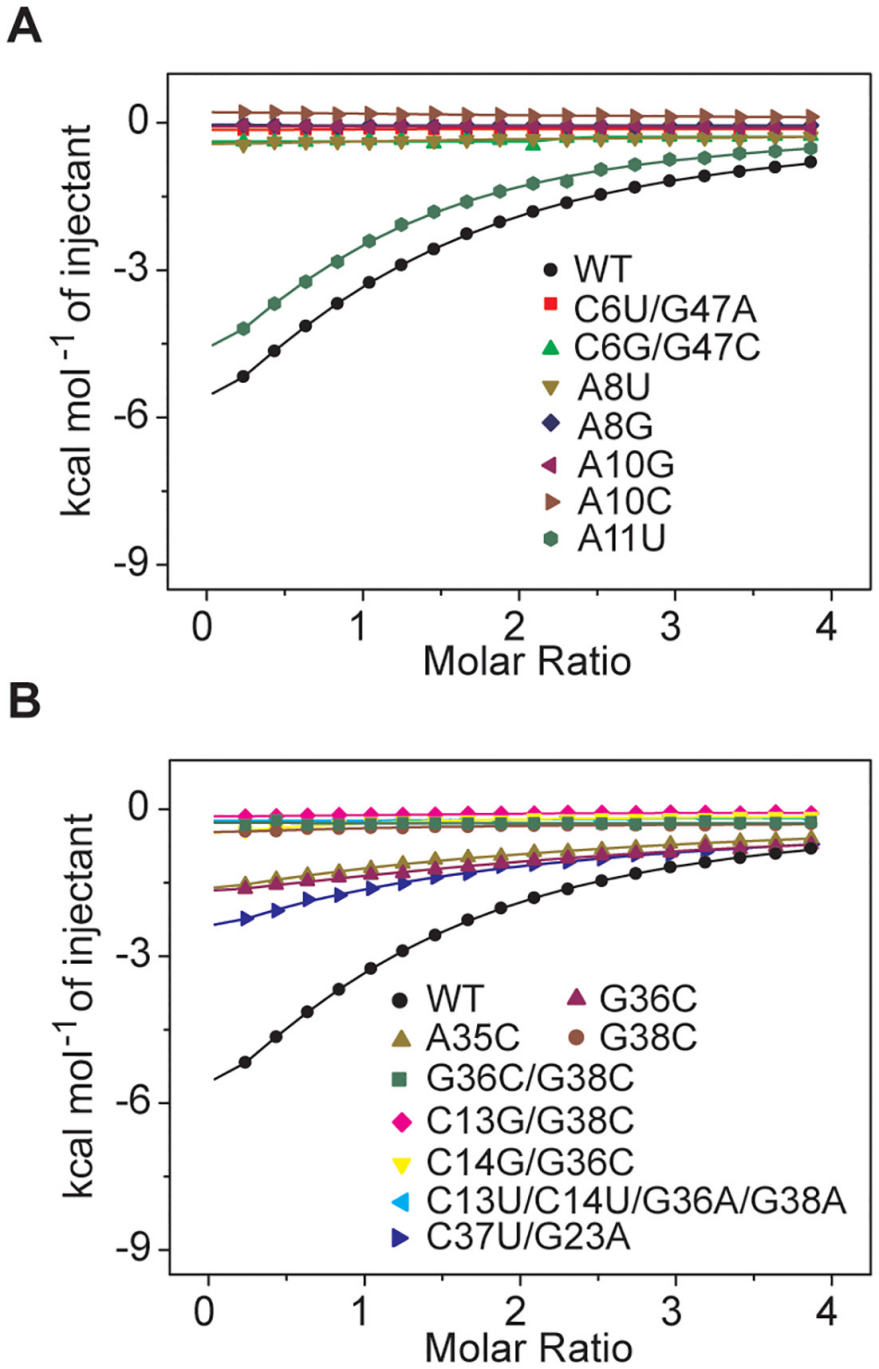
ITC to analyze the binding capability of NAD^+^ riboswitch domain 1 and a series of mutants. (**A**) Overlay of integrated fitted heat plots obtained by ITC experiments of WT domain 1 in comparison with mutants concerning nucleotides in the binding pocket. (**B**) Same as A but with mutants concerning nucleotides involved in the long-distance J1/J2 interactions as well as in junction J2.

The NAD^+^ riboswitch recognizes the ADP moiety of NAD^+^ by intense minor groove H-bond interaction with the base pair C6-G47 (Figure 2C–H). It is therefore not surprising that the alteration of the hydrogen donor/acceptor pattern by replacement of C6-G47 with U6-A47 and G6-C47, respectively, abolishes binding under otherwise the same conditions, analyzed by ITC (Figure 4A). Likewise, the A8•A7-U46 base triplet which stacks on the NAD^+^ adenine base is highly sensitive and mutation of A8 to either U8 or G8 is also not tolerated (Figure 4A). Furthermore, the nucleobase of A10 which is innersphere coordinated to a Mg^2+^ ion (M2) via its N7 atom (Figure 2K–M) is crucial for shaping the binding pocket and mutation of A10 to either G or C results in loss of binding as well. Distinct to all other nucleotides mentioned above, A11 is not involved in any tertiary interactions and directed outwards from the junction. As expected, the A11U mutant is tolerated and the binding activity to NAD^+^ remains comparable to the wild-type (WT) construct.

The long-distance pairing and the pronounced stacking interactions in junctions J1 and J2 seem crucial for creating the high affinity binding pocket of domain 1 for NAD^+^. The single point mutations G36C and G38C, respectively, and the double mutation G36C/G38C impair the interactions between J1 and J2, and all three mutants do not bind NAD^+^ under otherwise the same conditions. Additionally, we analyzed the crucial J1/J2 interaction for a possible tolerance of compensatory base pair mutations, such as C14G/G36C, C13G/G38C or C13U/C14U/G36A/G38A. However, these mutants have no affinity to NAD^+^ under otherwise the same conditions (Figure 4B). Moreover, A35C that changes the non-canonical base pair A35-G24 (J2) to canonical C35-G24, as well as the compensatory base pair mutation C37U/G23A (J2) also resulted in loss of the binding (Figure 4B). These results are consistent with phylogenetic sequence analysis revealing that the base sequences of junctions J1 and J2 are highly conserved (Supplementary Figure S1B).

### Mg^2+^-dependent folding and ligand recognition of domains 1 and 2

**Figure 5.**
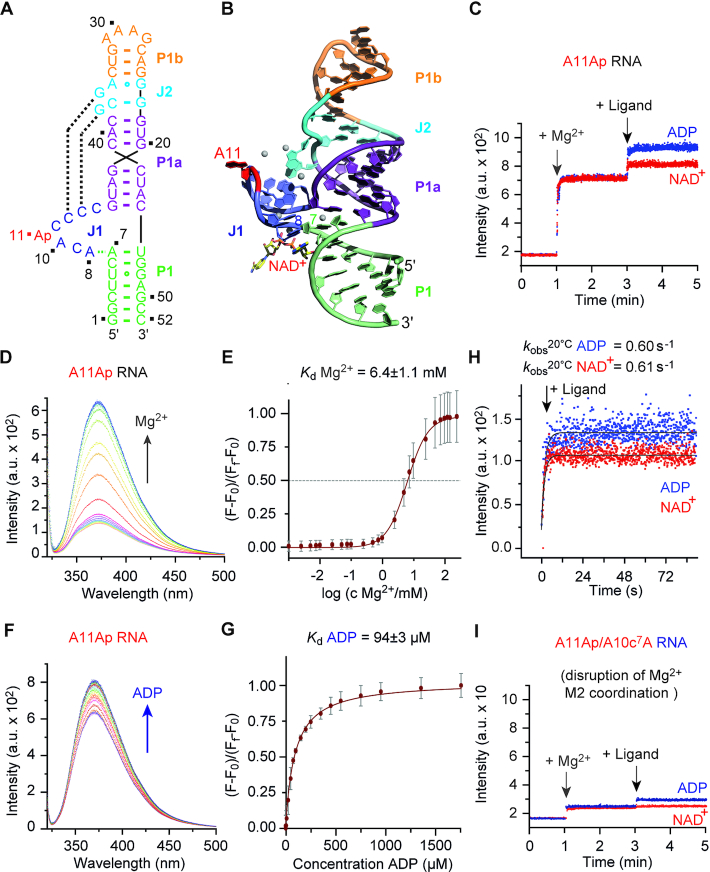
Fluorescence spectroscopic assessment of domain 1 folding and ligand binding. (**A**) Sequence and secondary structure of the 2-aminopurine (Ap) modified RNA used for fluorescence spectroscopic experiments. (**B**) The nucleoside A11 (in red) that was selected for Ap replacement is located close to the binding pocket and directed outwards. (**C**) Real time fluorescence traces for A11Ap domain 1 (*c* = 0.5 μM), upon Mg^2+^ (50 mM) and ligand (0.3 mM) additions. (**D**) Fluorescence changes upon titration of A11Ap domain 1 (*c* = 0.5 μM) with increasing concentrations of Mg^2+^ ions; fluorescence emission spectra (λ_ex_ = 308 nm) from 320 to 500 nm of the A11Ap variant for each Mg^2+^ concentration. (**E**) Normalized fluorescence intensity of the A11Ap variant plotted as a function of Mg^2+^ concentration. The graph shows the sigmoid fit (see ‘Materials and Methods’ section). Changes in fluorescence (F-F_0_) were normalized to the maximum fluorescence measured in saturating concentrations of Mg^2+^ ions. The obtained *K*_d_ value for Mg^2+^ in 50 mM KMOPS buffer, pH 7.5, 100 mM KCl, at 293 K, is indicated. (**F**) Fluorescence changes upon titration of A11Ap domain 1 (*c* = 0.5 μM) with ADP ligand; fluorescence emission spectra (λ_ex_ = 308 nm) from 320 to 500 nm of the A11Ap variant for each ADP concentration. (**G**) Normalized fluorescence intensity of the A11Ap variant plotted as a function of ADP concentration. The graph shows the best fit to a single site binding model (see ‘Materials and Methods’ section). Changes in fluorescence (F-F_0_) were normalized to the maximum fluorescence measured in saturating concentrations of ADP ligand. The obtained *K*_d_ value for ADP in 50 mM KMOPS buffer, pH 7.5, 100 mM KCl, 50 mM MgCl_2_, at 293 K, is indicated. (**H**) Monitoring the kinetics of domain 1 complex formation using the A11Ap labeled RNA. Exemplary fluorescence traces for NAD^+^ and ADP additions are depicted; conditions: 0.5 μM RNA, 100 mM KCl, 50 mM MOPS, pH 7.5, 293 K. Ligands: 20 mM MgCl_2_, 300 μM NAD^+^ or ADP. (**I**) Real time fluorescence traces for A10c^7^A modified A11Ap domain 1 (*c* = 0.5 μM), upon Mg^2+^ (50 mM) and ligand (0.3 mM) additions.

**Figure 6.**
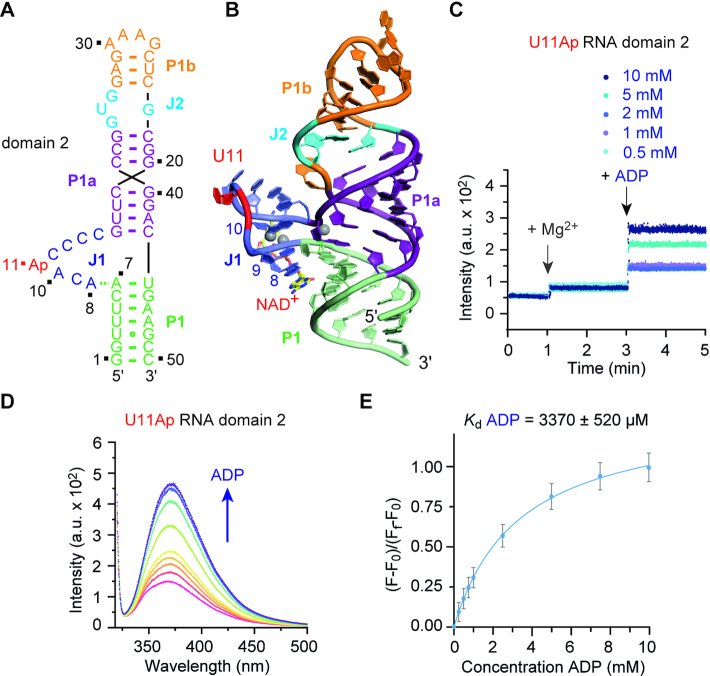
Fluorescence spectroscopic assessment of domain 2 folding and ligand binding. (**A**) Sequence and secondary structure of the 2-aminopurine (Ap) modified RNA used for fluorescence spectroscopic experiments. (**B**) The nucleoside U11 (in red) is structurally equivalent to A11 in domain 1 and was selected for Ap replacement. This allows direct comparison of the two domains. (**C**) Real time fluorescence traces for U11Ap domain 2 (*c* = 0.5 μM), upon Mg^2+^ (50 mM) and ADP additions at increasing concentrations (up to 10 mM). (**D**) Fluorescence changes upon titration of A11Ap domain 2 (*c* = 0.5 μM) with ADP ligand; fluorescence emission spectra (λ_ex_ = 308 nm) from 320 to 500 nm of the A11Ap variant for each ADP concentration. (**E**) Normalized fluorescence intensity of the A11Ap variant plotted as a function of ADP concentration. The graph shows the best fit to a single site binding model (see ‘Materials and Methods’ section). Changes in fluorescence (F-F_0_) were normalized to the maximum fluorescence measured in saturating concentrations of ADP ligand. The obtained *K*_d_ value for ADP in 50 mM KMOPS buffer, pH 7.5, 100 mM KCl, 50 mM MgCl_2_, at 293 K, is indicated.

With the crystal structures of both domains in hands, we set out to shed light on possible distinctions in folding and ligand recognition between the two domains. We therefore performed fluorescence measurements on a site-specifically labeled 2-aminopurine (Ap) riboswitch variant (Figures 5A and 6A). From the structures, it is obvious that A11 (domain 1) (Figure 5B) and U11 (domain 2) (Figure 6B) of junction 1 are not involved in any tertiary interactions. They are unstacked and solvent-exposed in the ligand-bound state and we therefore considered Ap replacements at position 11 ideal to monitor Mg^2+^ and/or ligand-induced folding. Indeed, following the fluorescence response over time, the addition of Mg^2+^ ions results in a pronounced fluorescence increase (Figure 5C) for domain 1, consistent with the local conformational change of Ap11 into an extrahelical position. Subsequent addition of NAD^+^ or ADP provides a further, yet smaller fluorescence increase, consistent with a slight conformational adaption of the RNA junction upon ligand binding (Figure 5C). Importantly, the concentration-dependent fluorescence response data of Mg^2+^ titrations (Figure 5D) were fit to sigmoid function and gave an apparent dissociation constant, *K*_D_^Mg^ of 6.4 ± 1.1 mM (Figure 5E). In principle, the Ap fluorescence assay also allows the determination of ligand affinities, however, because of spectral interference with NAD^+^ and 2-aminopurine at high NAD^+^ concentrations, the determination was reliable for the ADP ligand only. At saturating Mg^2+^ concentrations, the concentration-dependent fluorescence response data of ADP titrations (Figure 5F) were fit to a single-site binding model and resulted in an apparent dissociation constant, *K*_D_^ADP^ of 94±3 μM (Figure 5G). We were furthermore wondering if the Mg^2+^ ion M2 which is innersphere coordinated to N7 of A10 and the 5′ phosphates of C9 and A8, is critical for proper pre-folding of the A10-C9-A8 turn in the pocket (Figure 2L). Indeed, addition of Mg^2+^ in saturating concentrations results in a very minor increase of Ap fluorescence of the A10c^7^A/A11Ap mutant and remained nearly unaltered upon NAD^+^ (or ADP) addition (Figure 5I). We conclude that because of the inability to coordinate the Mg^2+^ ion (M2), the required conformation of the binding pocket does not properly pre-fold to enable the ‘lock and key’-type recognition as observed for the WT domain 1. Furthermore, we note that the open binding pocket is rather untypical for riboswitches which usually encapsulate their ligands through relatively slow ligand-induced and RNA-adaptive recognition processes. Ligand recognition of the NAD^+^ riboswitch was estimated to be in the order of one per second (Figure 5H) which is rather fast and also consistent with an open and well-accessible, pre-folded binding site.

Distinct to domain 1, the junction J1 of the binding pocket of domain 2 remains dynamic and conformationally largely undefined in the absence of ligand, even at very high Mg^2+^ ion concentrations. This is obvious from the marginal fluorescence increase of the U11Ap mutant of domain 2 in response to Mg^2+^ (Figure 6). Importantly, when we titrated the U11Ap mutant by the potential ligand ADP of increasing concentrations up to 10 mM into a 50 mM Mg^2+^ containing buffer, a significant fluorescence increase was observed (Figure 6C), which is consistent with ADP-induced adaptive recognition and ADP binding to domain 2. When the concentration-dependent fluorescence response data of the titrations (Figure 6D) are fit to a single-site binding model and an apparent dissociation constant *K*_D_^ADP^ of 3.4 ± 0.5 mM is obtained (Figure 6E), reflecting a 35-fold weaker binding of domain 2 compared to domain 1.

Taken together, the fluorescence spectroscopic studies reveal that the J1/J2 long-range interaction through Watson–Crick base pairing is the crucial determinant to tune the ligand binding affinity. Although we already knew from ITC measurements that the compensatory base-pair mutations C13U/C14U/G36A/G38A in domain 1 resulted in a loss of affinity to NAD^+^, we independently confirmed this finding by the Ap fluorescence assay (Supplementary Figure S13). Moreover, we were wondering if the loss in affinity results from the decrease in pairing strengths (two GC versus two AU base pairs) or if stacking of the four cytosines (C12-C13-C14-C15) is the more critical factor for pre-folding of the binding pocket. In the C13U/C14U/G36A/G38A mutant, the C_4_ sequence in J1 is altered to CU_2_C which might be inferior in forming the pyrimidine staple. Therefore, we synthesized the G36A/G38A/A11Ap mutant of domain 1, which retains the possibility for intact stacking of the four Cs in J1 but disrupts the long-range GC base pair interaction; we found that this mutant indeed binds ADP and NAD^+^ to a significant extent (Supporting Figure S13). This finding is consistent with the fact that the G36A/G38A domain 1 mutant is essentially domain 2, lacking the long-range interaction but leaving the conserved nucleotides in J1 and their stacking identities absolutely unchanged.

### Comparison to NAD^+^ riboswitch domain 1 from *Candidatus koribacter versatilis*

We note that our structures of the NAD^+^ riboswitch domain 1 from *Acidobacterium capsulatum* and from an environmental sequence of the *nadA* RNA motif are consistent with the recent structure of domain 1 from *Candidatus koribacter versatilis* (21).

### Concluding remarks

The NAD^+^ sensitive *nadA* RNA motif is intriguing because its consensus sequence model carries the features of a tandem aptamer architecture (20). However, while the first domain has been well characterized by biochemical and crystal structure analyses early on (20,21), the structure and function of the second domain remained murky. In particular, the nature of the cognate ligand of domain 2 remained unclear because chemical probing experiments revealed ligand-induced structural modulations only for the first domain while none were observable for the second domain (20). This is surprising for several reasons: first, bioinformatics analysis demonstrated that both aptamer domains always reside in tandem in the *nadA* RNA motif and both are required for maximal gene regulation (20). Second, the two domains are relatively similar and this suggests that they might bind two separate, but identical ligands, as observed for instance for the tandem aptamers in glycine riboswitches that cooperatively bind two separate glycine molecules (9–13) or for some TPP riboswitches (29). Third, the original *in vitro* assays were consistent with NAD^+^ bound with an apparent 1:1 stoichiometry, and with *K*_D_ values that approached 100 μM (14). This binding was attributed entirely due to recognition of the 5′-ADP moiety of the cofactor by the first aptamer domain (20,21). The fact that only the 5′-ADP moiety of NAD^+^ was recognized, and ADP or ATP alone are bound with similar affinities, leaves room for discussions about the true nature of the cognate ligand. Consequently, it was speculated that domain 2 might recognize the other part of the NAD^+^ ligand, namely the NMN moiety, and hence could increase cellular specificity for NAD^+^ (20). Most supportive for NAD^+^ being the cognate ligand is the genomic context which finds the tandem *nadA* motif never associated with genes unrelated to NAD^+^ biosynthesis, and also not in combination with genes related to ADP or ATP biosynthesis (20).

In the present work, we now show that domain 2 selectively binds to ligands carrying an adenosine 5′-diphosphate (5′-ADP) moiety, including NAD^+^. The interactions in the binding pocket with the ligand are the same as found for domain 1. However, its affinity to the ligands is about 35-fold lower. The structural reason for this observation is the missing, shifted or distinct sequence of the second bulge J2 so that the crucial long-distance interaction to J1 cannot form any more. This structural feature also seems responsible that proper folding of the binding pocket cannot be induced by Mg^2+^ alone, and consequently cannot offer a ‘lock-and-key'-type recognition mode as encountered for domain 1. Instead, much higher ligand concentrations under otherwise same Mg^2+^ concentrations are required for the adaptive recognition process of the same ligand by domain 2.

**Figure 7.**
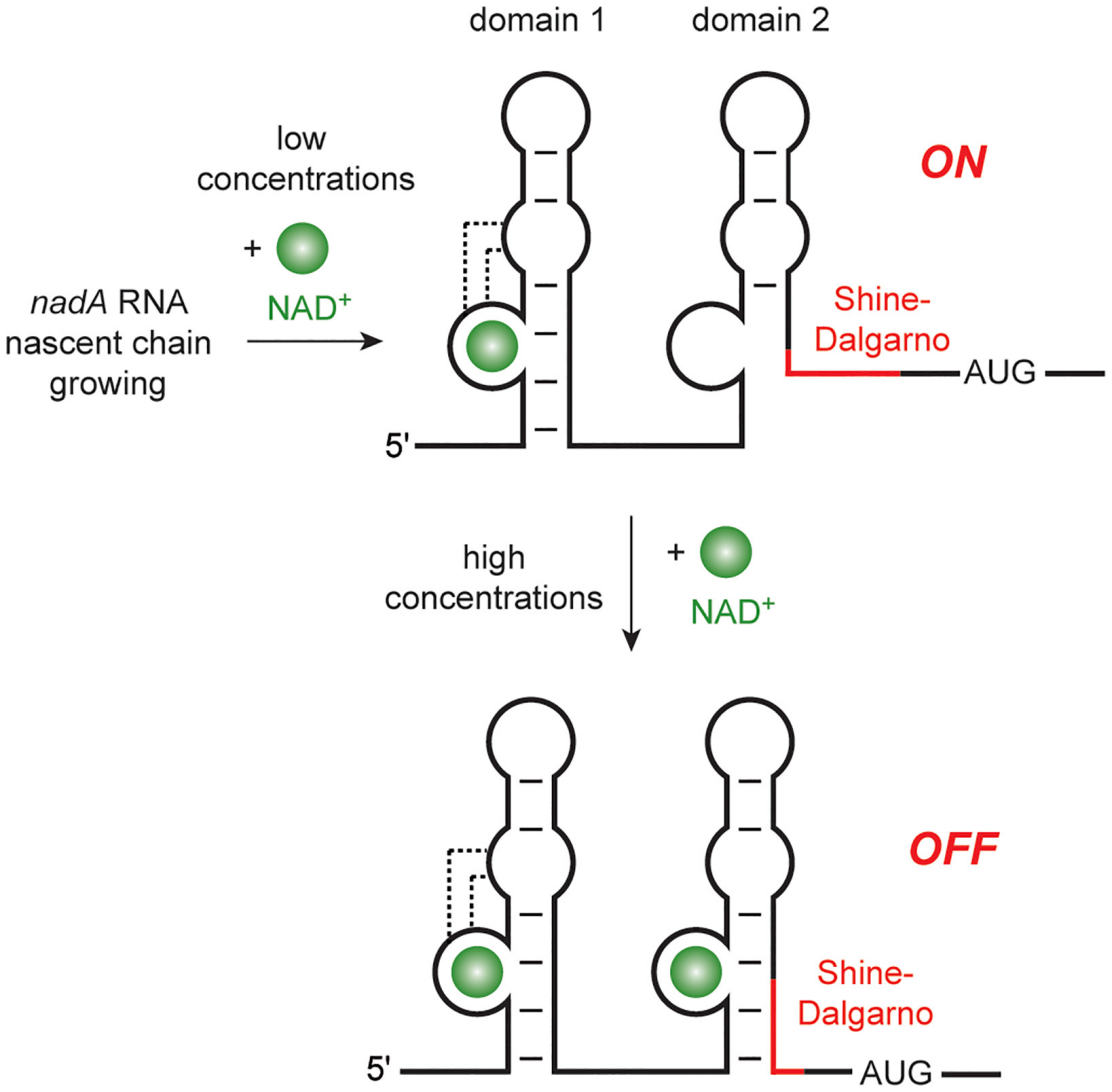
Putative gene regulation mechanism of *nadA* RNA riboswitches. A mechanistic role of the tandem architecture is proposed in which the OFF signal of the translational machinery primed at low ligand concentrations (domain 1), and eventually triggers repression of translation by sequestration of the ribosomal binding site (Shine-Dalgarno) in P1 as soon as millimolar ligand concentrations are reached in the cell (domain 2).

Domain 2 contains the putative regulatory helix (P1) that sequesters the ribosomal binding site and therefore has been proposed as the determinant which is responsible for providing the ON versus OFF signal for translation. Considering the very high concentrations of NAD^+^ in bacterial cells, which have been reported to be 2.6 mM or even higher (30,31), a scenario in which NAD^+^ triggers the gene response via the second domain appears possible (Figure 7). If so, then remains the open question on the function of the first domain with significantly higher affinity. Activity of a high affinity aptamer at first during growing of the nascent RNA certainly structures the leader sequence and might be a handle to time (i.e. delay) the actually response with precision based on the sequential folding path. It is conceivable that a mechanism of sensing low concentrations might prime and stall the response machinery for triggering the actual OFF signal once the high final concentrations have been reached from NAD^+^ biosynthesis (Figure 7).

Although a two-concentration sensing riboswitch might be advantageous for the time course of regulation of high-abundant metabolite biosynthesis, we should be cautious with ultimate final conclusions. At this time, it cannot be ruled out that a distinct, thus far undiscovered high-affinity ligand might exist for domain 2. We remind of the *ydaO* motif RNA which was reported to weakly bind ATP (32), and later on turned out to selectively respond to c-di-AMP with subnanomolar affinity (33). It also cannot be ruled out completely that the second domain might assist in recognition of the NMN moiety of NAD^+^. The dimerization of individual domains we observe in the crystals might feed this impression. Nevertheless, we favor the view that the two domains act independently from each other because in solution, monomers of the individual domains are observed exclusively and the dimerization interactions observed in the crystals are distinct for the three crystal constructs (domain 1 GAAA, domain 1–U1A and domain 2 GAAA) and appear to be weak. To this end, we also note that all our efforts to crystallize the full-length (two-domain) riboswitch failed. Finally, what makes it difficult to narrow down possible mechanistic scenarios of this riboswitch further is that—despite intensive efforts—we were unsuccessful to establish a robust cellular system in *E. coli* to analyze the gene response of the *nadA* WT RNA motif and diverse mutants, which was mainly due to the high concentrations of NAD^+^ needed in the broth medium that negatively affected cell growth.

Coming back to the question where the specificity for NAD^+^ of this riboswitch comes from, we mention that the ligand assignment has been merely based on the genomic context which finds the tandem *nadA* motif never associated with genes unrelated to NAD^+^ biosynthesis, and also not in combination with genes related to ADP or ATP biosynthesis (20). Furthermore, it is conceivable that RNA *structural* discrimination to generate aptamer specificity for NAD^+^ over ATP or ADP might not be required in the cell because the cellular concentrations of NAD^+^ are higher than those of ATP, and significantly higher than those of ADP (30,31).

Taken together, our work contributes to a significantly increased understanding of possible roles of the NAD^+^ riboswitch tandem motif for gene regulation. In particular, the finding that domain 2 is sensitive for NAD^+^ at high concentrations and that domain 2 applies the identical stacking and hydrogen bonding network for Mg^2+^-mediated ligand recognition as domain 1 does, makes the function as a two-concentration sensor module for the same ligand reasonable (Figure 7). We therefore propose a putative mechanistic role for a time-controlled signal that is gear-up but retarded at low ligand concentrations (domain 1), and eventually triggers repression of translation by sequestration of the ribosomal binding site in P1 as soon as millimolar ligand concentrations are reached in the cell (domain 2).

## DATA AVAILABILITY

Atomic coordinates and structure factors for the reported crystal structures have been deposited with the Protein Data Bank under accession numbers 7D7V (17delU1A-NAD^+^structure), 7D7W (18GAAA-NAD^+^ structure), 7D7X (18GAAA-ADP structure), 7D7Y (18GAAA-ATP structure), 7D7Z (18GAAA-NAD-Mn^2+^ soaking structure), 7D81 (832GAAA-NAD^+^ structure) and 7D82 (832GAAA-NAD^+^-Mn^2+^ soaking structure).

## Supplementary Material

gkaa1029_Supplemental_Files
