# Supplementary material for: Structural distinctions between NAD+ riboswitch domains 1 and 2 determine differential folding and ligand binding
Source: Nucleic Acids Res. 2020 Nov 10;48(21):12394–406. doi: 10.1093/nar/gkaa1029 (PMC7708056; doi:10.1093/nar/gkaa1029)
Supplement: gkaa1029_Supplemental_Files [file gkaa1029_supplemental_files.zip › Supplementary Materials_gkaa1029 corrected proofs.pdf]

Supplementary Materials

Structural distinctions between NAD^+ riboswitch domains 1 and 2 determine differential folding and ligand binding

Hao Chen[1,#], Michaela Egger[2,#], Xiaochen Xu[1, #], Laurin Flemmich[2], Olga Krasheninina[2], Aiai Sun[3], Ronald Micura[2,*], and Aiming Ren[1,*]

[1]Life Sciences Institute, Zhejiang University, Hangzhou, Zhejiang 310058, China

[2]Institute of Organic Chemistry, Center for Molecular Biosciences Innsbruck, University of Innsbruck, Innsbruck, 6020, Austria

[3]School of Chemistry and Materials Science, Hangzhou Institute for Advanced Study, University of Chinese Academy of Sciences, 1 Sub-lane Xiangshan, Hangzhou 310024, China

Contents

Supplementary Figures S1-S13 ………………………………………………………… 2

Supplementary Tables S1-S3 ………………………………………………………..…16

Supplementary Figures S1-S13

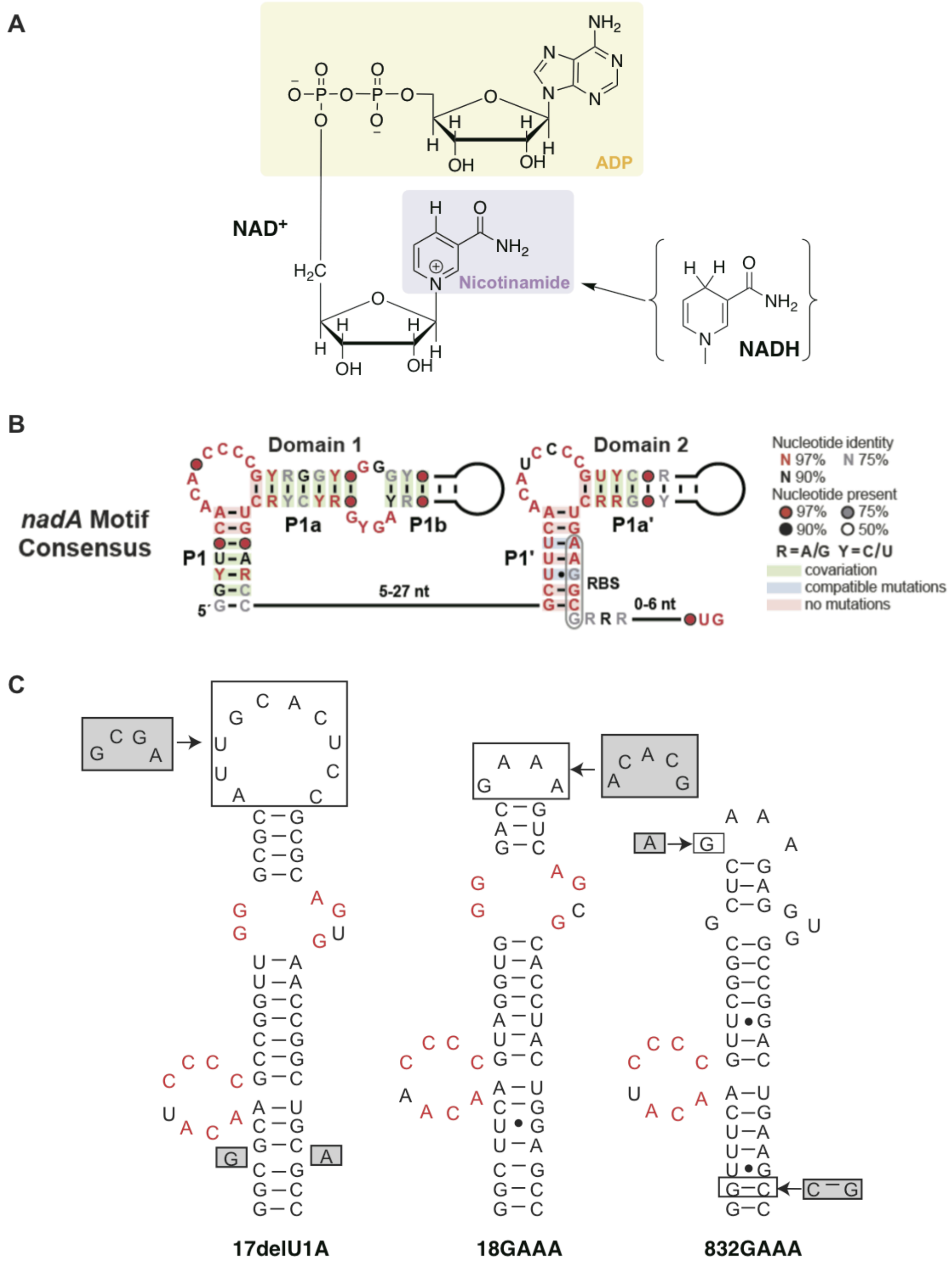

Supplementary Figure S1. *nadA* riboswitch motif. (**A**) Chemical structure of NAD^+ and NADH. (**B**) *nadA* consensus motif illustration highly conserved nucleosides based on the phylogenetic sequence analysis [14]. (**C**) RNA constructs used for the crystallographic structure determination. Mutations made in non-conserved nucleoside regions are indicated by boxes (grey boxes display the wild-type sequence, white boxes display the sequence in the crystallized RNA; a deletion of the wt G-A pair in stem P1 was made in 17delU1A); see also Supplementary Table S1.

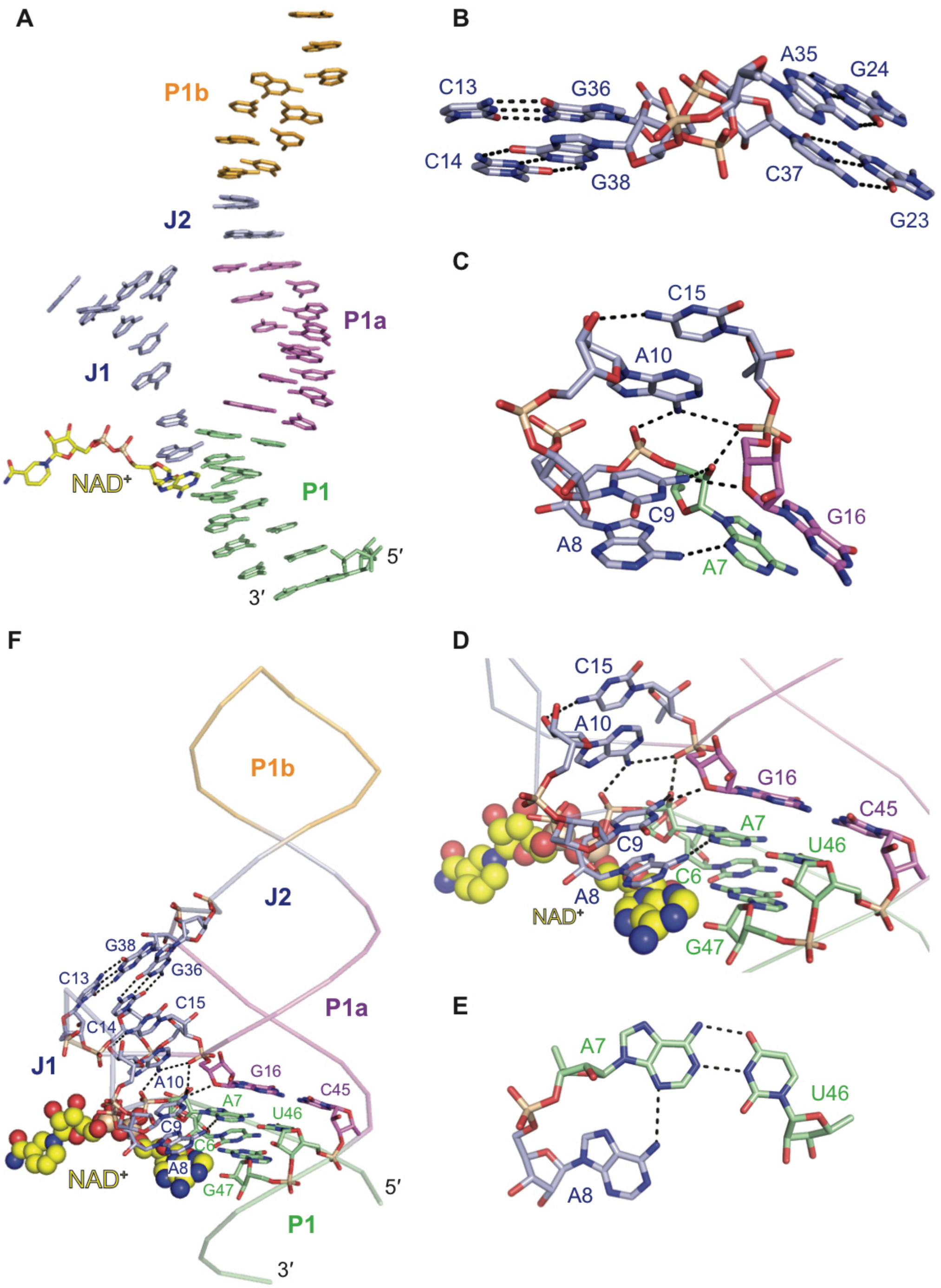

Supplementary Figure S2. Structure of 18GAAA-D1 domain 1 RNA. (**A**) Illustration of nucleobase stacking of all nucleotides in the structure. Nucleobases of J2 intercalate between stem P1a and P1b and form a long helix together with stem P1. Note that two bases of J1 form two long-distance base pairs with two bases of J2, and a base staple is built upon on the adenine moiety of ligand NAD$^+$. (**B**) Close-up view of the base pairing interactions between junctions J1 and J2. (**C**) Close-up view of the J1 interaction network that support coaxial stacking of P1 (A7) onto P1a (G16). (**D**) Close-up view of the interactions in the binding pocket. (**E**) Close-up view of the base triple that forms the ceiling of the binding pocket. (**F**) Overall view of the interactions in the binding pocket.

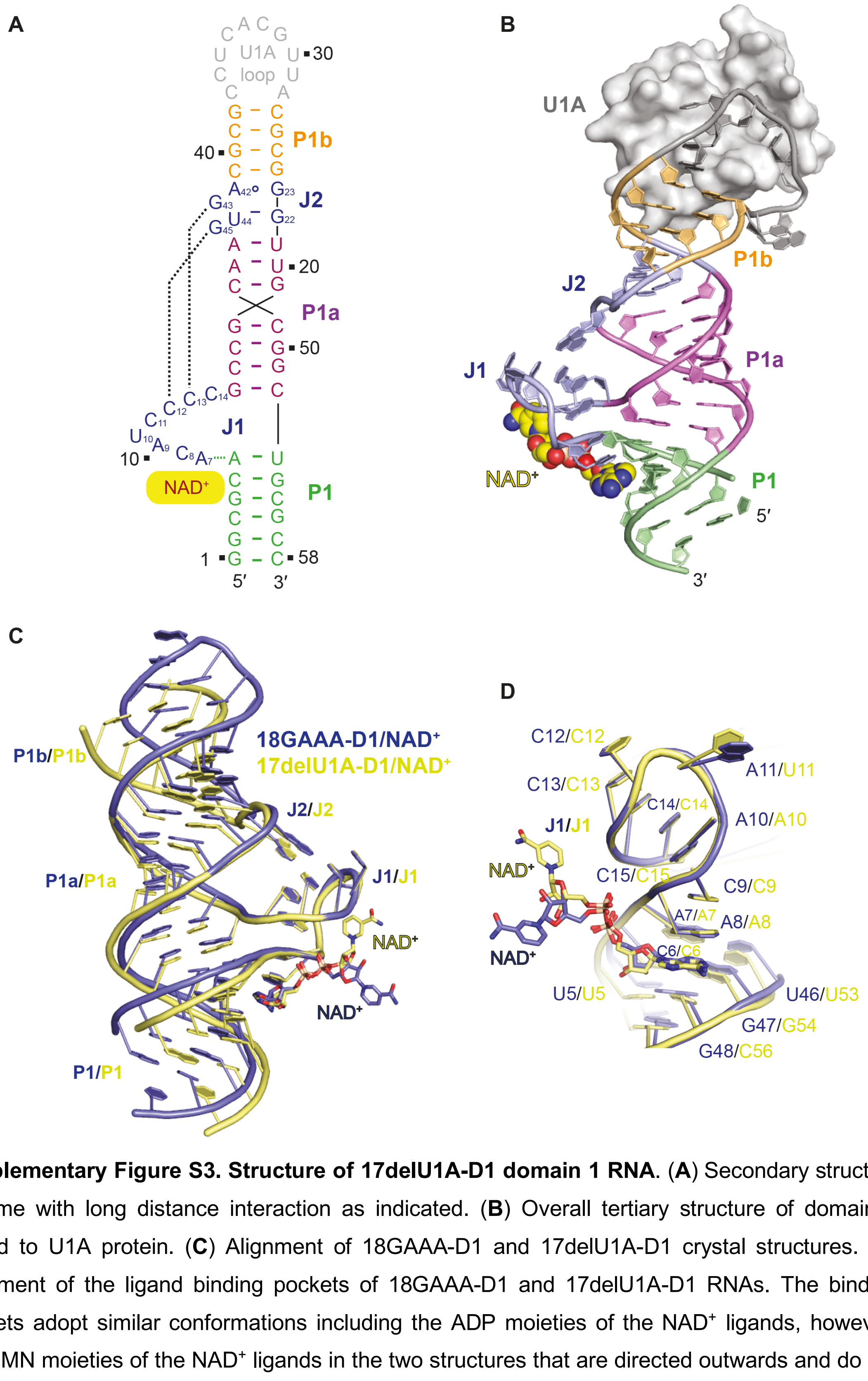

Supplementary Figure S3. Structure of 17delU1A-D1 domain 1 RNA. (**A**) Secondary structure scheme with long distance interaction as indicated. (**B**) Overall tertiary structure of domain 1 bound to U1A protein. (**C**) Alignment of 18GAAA-D1 and 17delU1A-D1 crystal structures. (**D**) Alignment of the ligand binding pockets of 18GAAA-D1 and 17delU1A-D1 RNAs. The binding pockets adopt similar conformations including the ADP moieties of the NAD$^+$ ligands, however, the NMN moieties of the NAD$^+$ ligands in the two structures that are directed outwards and do not interact with their pockets adopt distinct conformations.

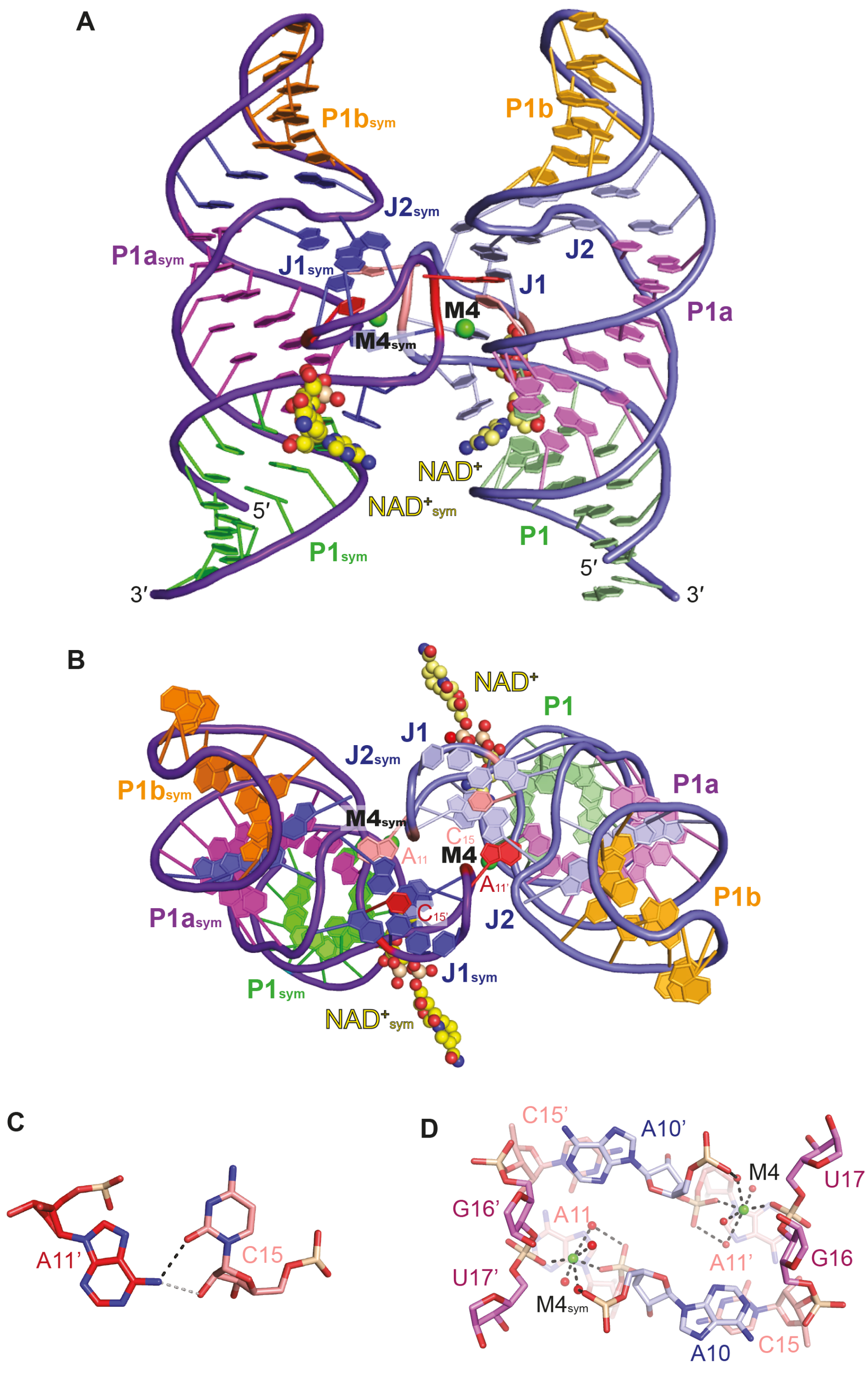

Supplementary Figure S4. Dimer formation of 18GAAA-D1 domain 1 RNA. (**A**) The 18GAAA-D1 RNA bound to NAD^+ forms homodimers in the crystal involving J1 and one cation (labeled M4) at the interface. (**B**) Same as (A) but view from the top. (**C**) Close-up view of the nucleotide interactions at the interface: C15 in J1 forms hydrogen bonds with A12' from J1 region of the symmetric molecule. (**D**) Close-up view of the cation interactions at the interface: One cation forms inner-sphere and outer-sphere coordination with pU16 (the phosphate between G16 and U17) and pA11', pA10' from the symmetric molecule.

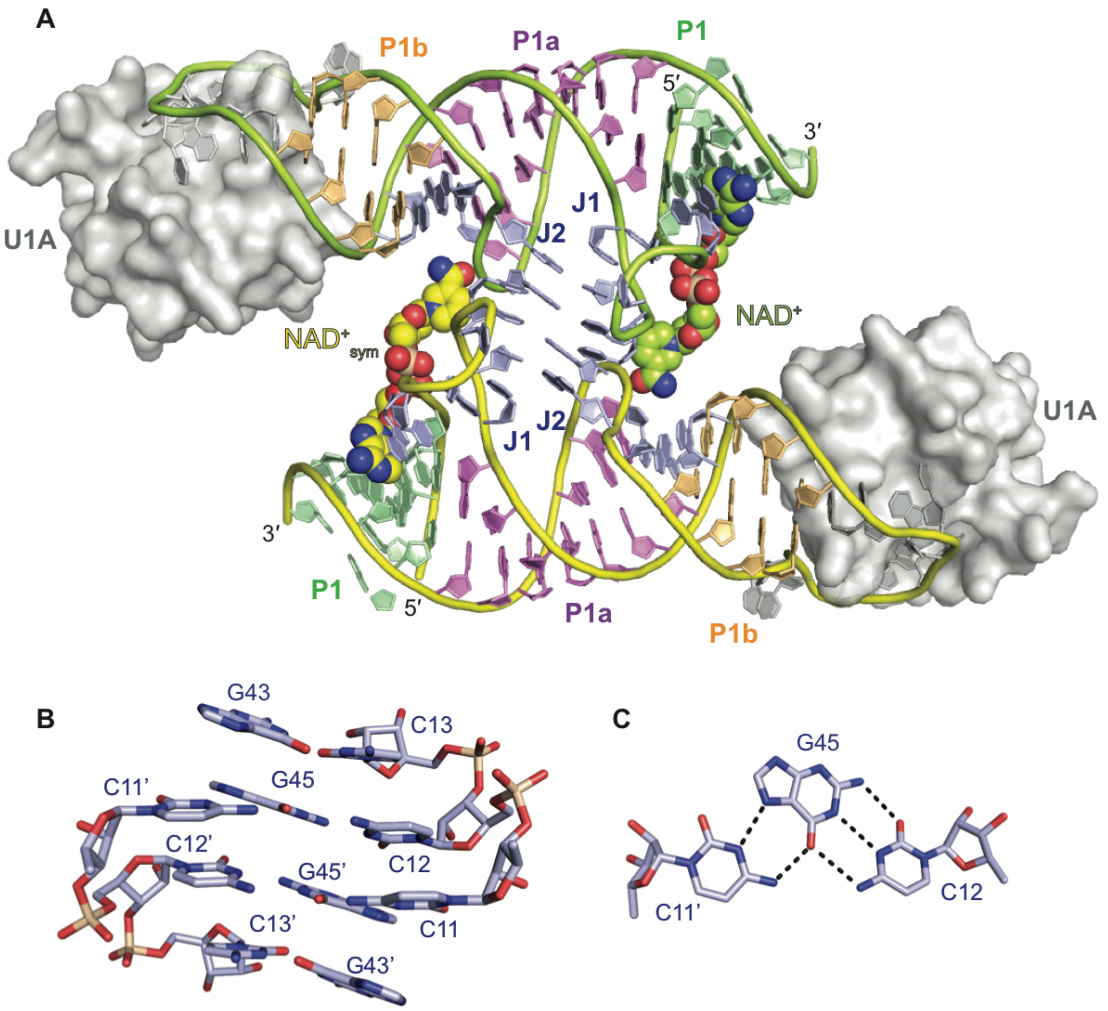

Supplementary Figure S5. Dimer formation of 17delU1A-D1 domain 1 RNA. (**A**) The 17delU1A-D1 bound to NAD$^+$ forms a homodimer in the crystal, in which both J1 and J2 are at the interface. (**B**) Close-up view of the nucleotide interactions at the interface: a base triple is formed between G45-C12 of one molecule and C11' of the symmetric molecule. (**C**) Close-up view of base triple C11'•G45-C12 at the interface.

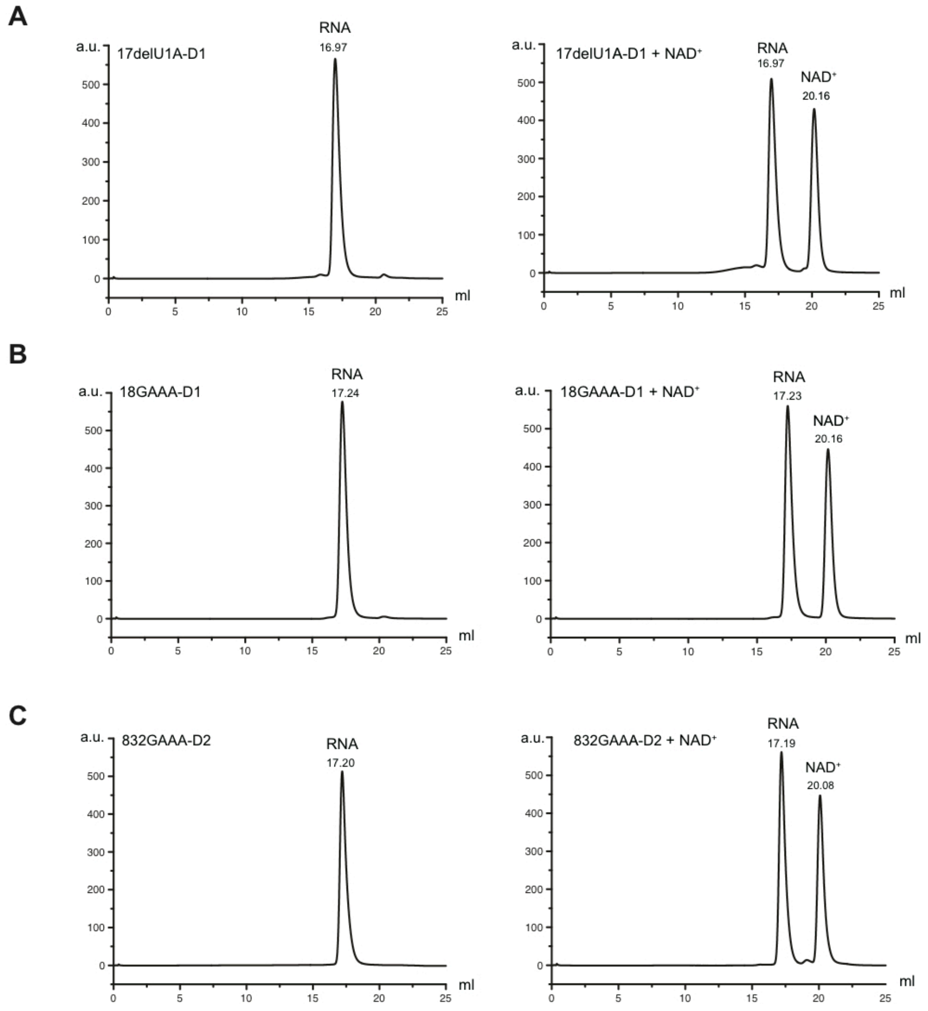

Supplementary Figure S6. Gel filtration analysis of 17delU1A-D1 domain 1, 18GAAA-D1 domain 1 and 832GAAA-D2 domain 2 RNAs. Chromatograms of 17delU1A-D1 (**A**), 18GAAA-D1 (**B**) and 832GAAA-D2 (**C**) RNA constructs and their NAD^+ complexes are consistent with their occurrence as monomers in solution. The size-exclusion experiments were run on the Superose^{TM} 6 Increase 10/300 GL column (GE Healthcare) that was pre-equilibrated with the buffer (50 mM HEPES, pH 7.0, 50 mM NaCl, 5 mM $MgCl_2$). Data was processed in Excel and Origin.

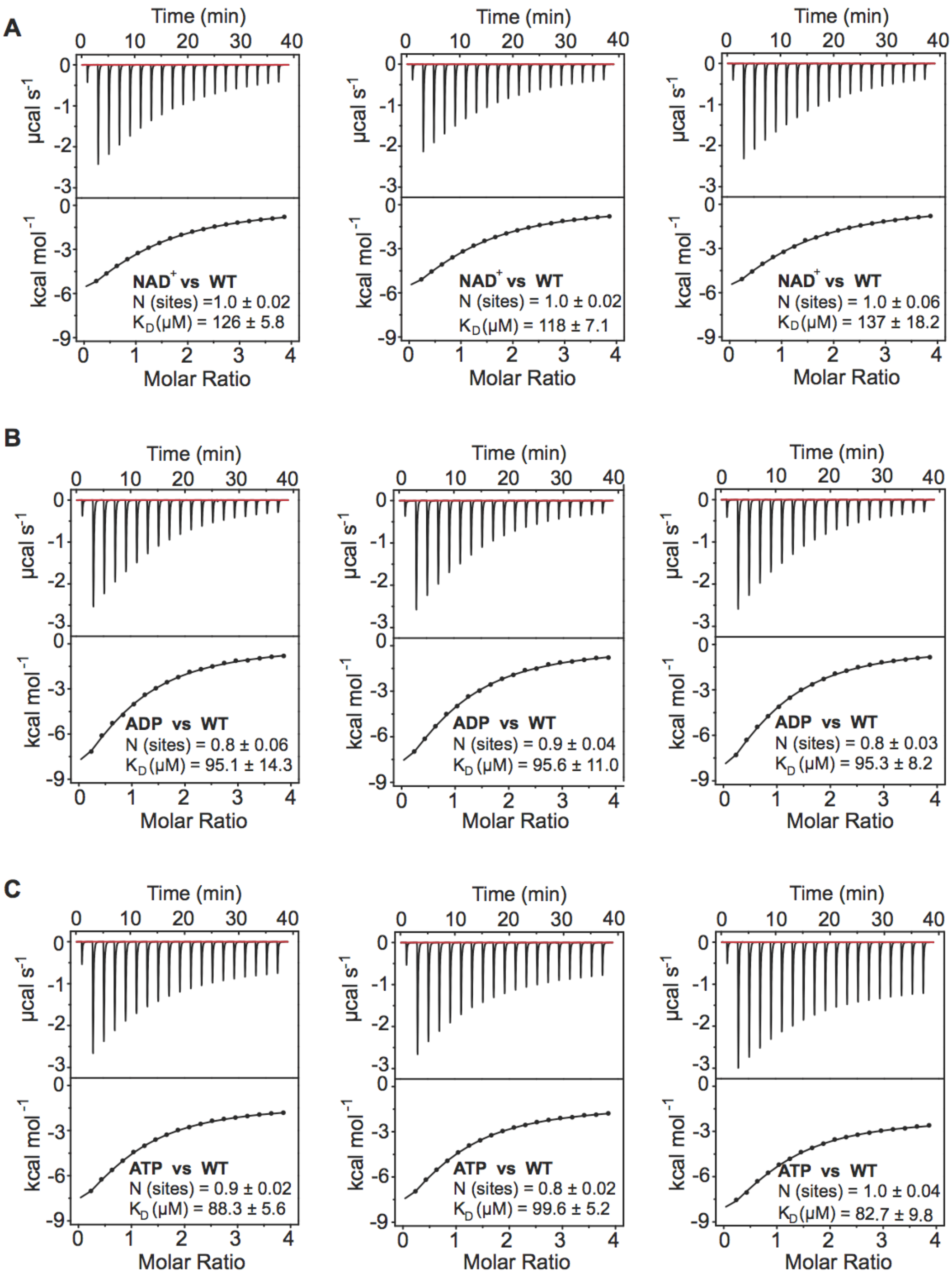

Supplementary Figure S7. Three independently repeated ITC titration experiments of 18GAAA-D1 domain 1 RNA with NAD$^+$ (A), ADP (B) and ATP (C). All titrations were performed at 20 °C in 50 mM HEPES pH 7.0, 50 mM NaCl, 10 mM $MgCl_2$. For c-values see Supplementary Table S3. The K_d and N parameters obtained from the fits are shown as text in the individual ΔH windows.

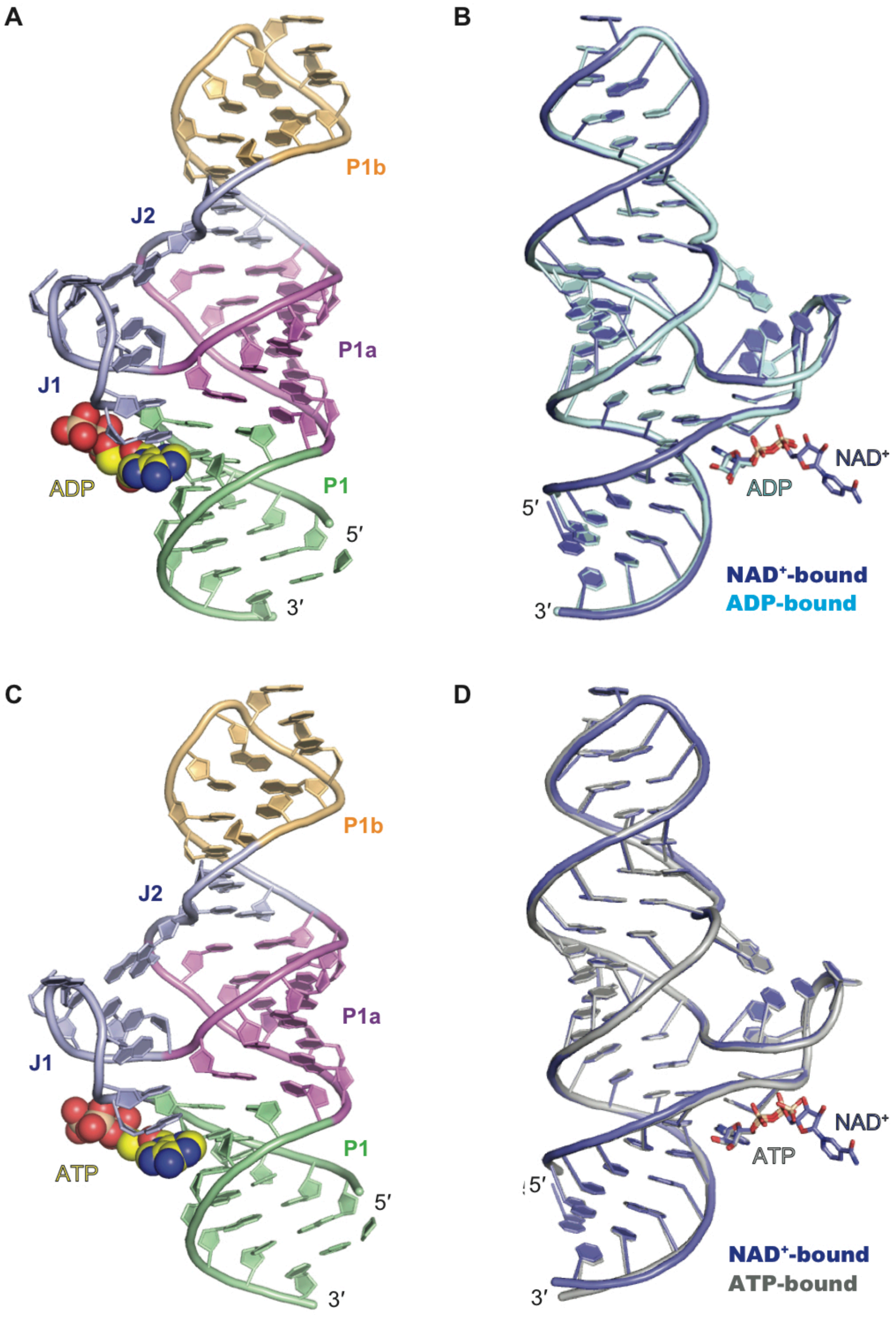

Supplementary Figure S8. Overall tertiary structures of 18GAAA-D1 domain 1 RNA in complex with ADP and ATP. (**A**) Tertiary structure domain 1 RNA in complex with ADP. (**B**) Alignment of NAD^+-bound and ADP-bound domain 1 RNA structures. (**C**) Tertiary structure of domain 1 RNA in complex with ATP. (**D**) Alignment of NAD^+-bound and ATP-bound domain 1 RNA structures.

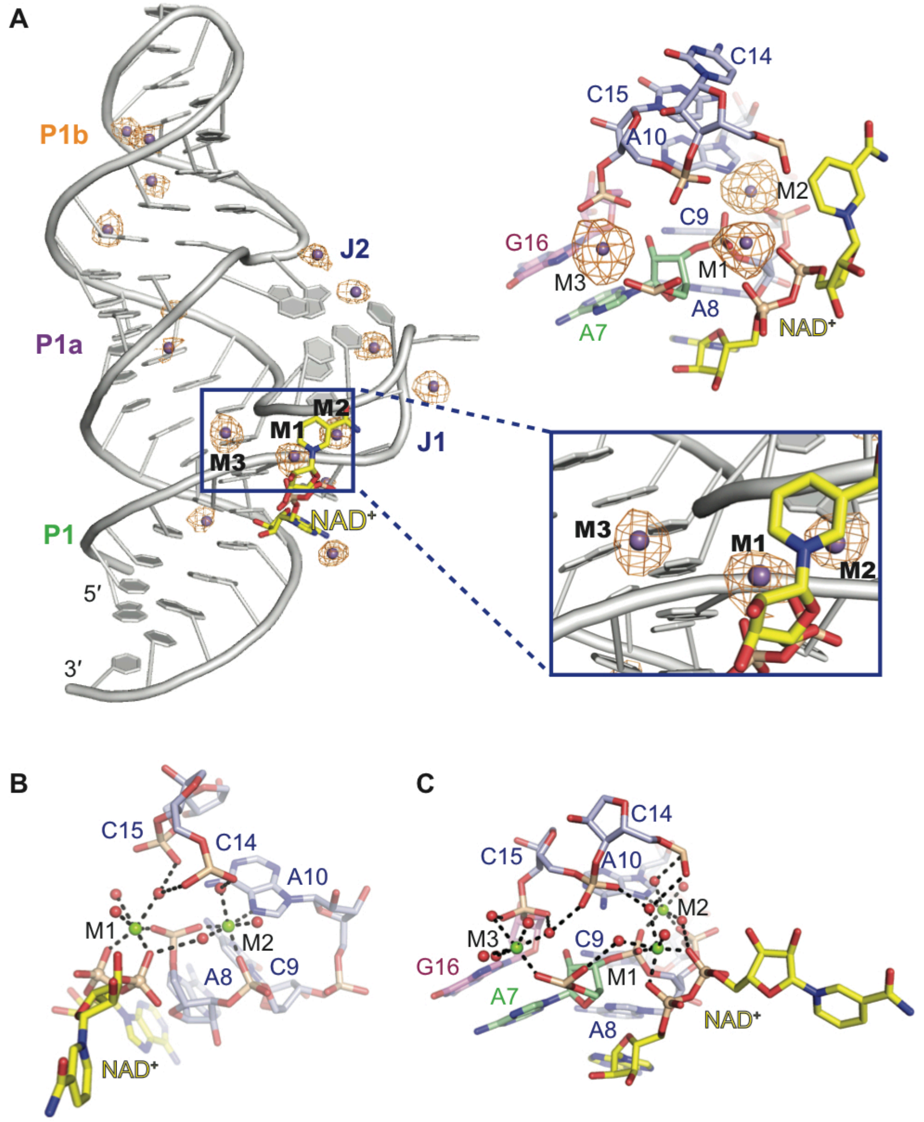

Supplementary Figure S9. Anomalous difference electron density map for Mn^{2+}-soaked NAD^{+}-bound 18GAAA-D1 domain 1 RNA crystals. (**A**) Anomalous electron density map for Mn^{2+} sites (purple balls) of NAD^{+}-bound 18GAAA-D1 RNA and close-up view of the three cation binding sites in the binding pockets. (**B**, **C**) The coordination network of the three Mg^{2+} cations in the binding pocket of 18GAAA-D1 domain 1 RNA visualized from different viewing angles.

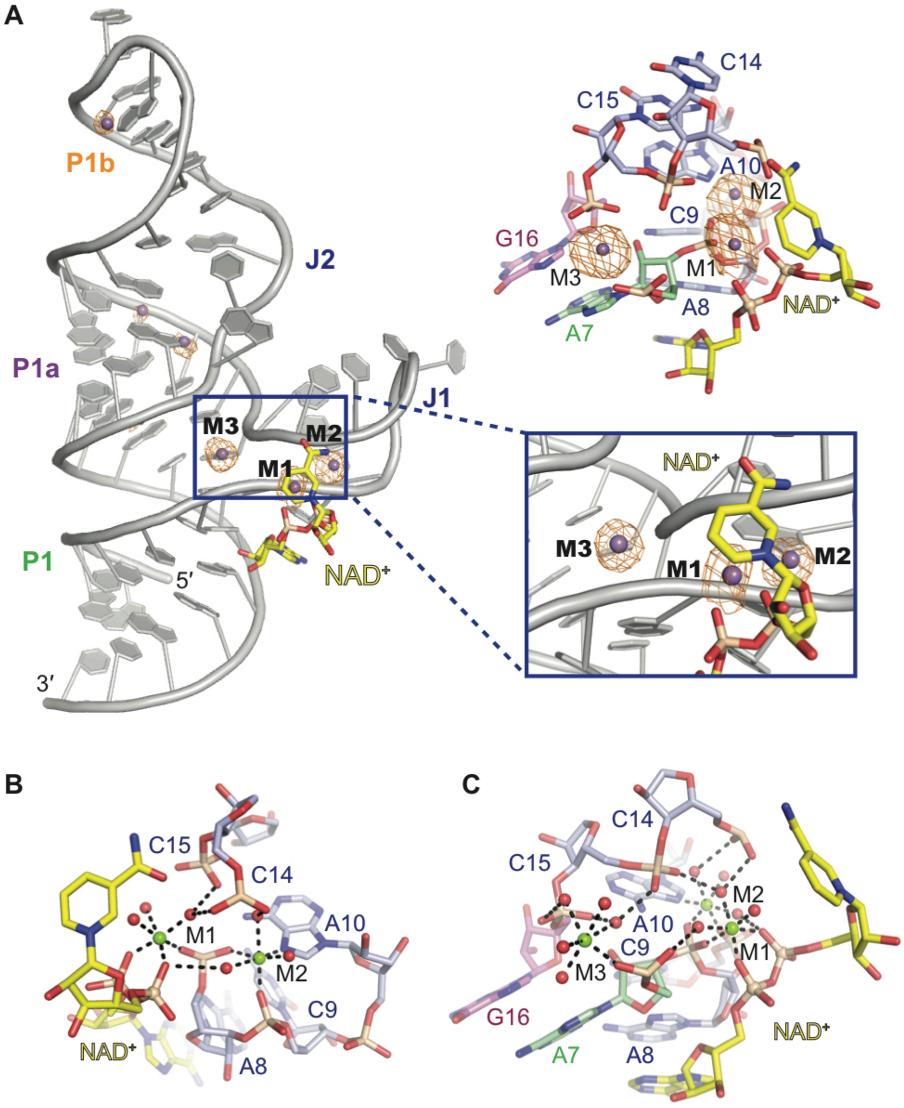

Supplementary Figure S10. Anomalous difference electron density map for Mn^{2+}-soaked NAD^{+}-bound 832GAAA-D2 domain 2 RNA crystals. (**A**) Anomalous electron density map for Mn^{2+} sites (purple balls) of NAD^{+}-bound 832GAAA-D2 RNA and close-up view of the three cation binding sites in the binding pockets. (**B**, **C**) The coordination network of the three Mg^{2+} cations in the binding pocket of 18GAAA-D1 domain 2 RNA visualized from different viewing angles.

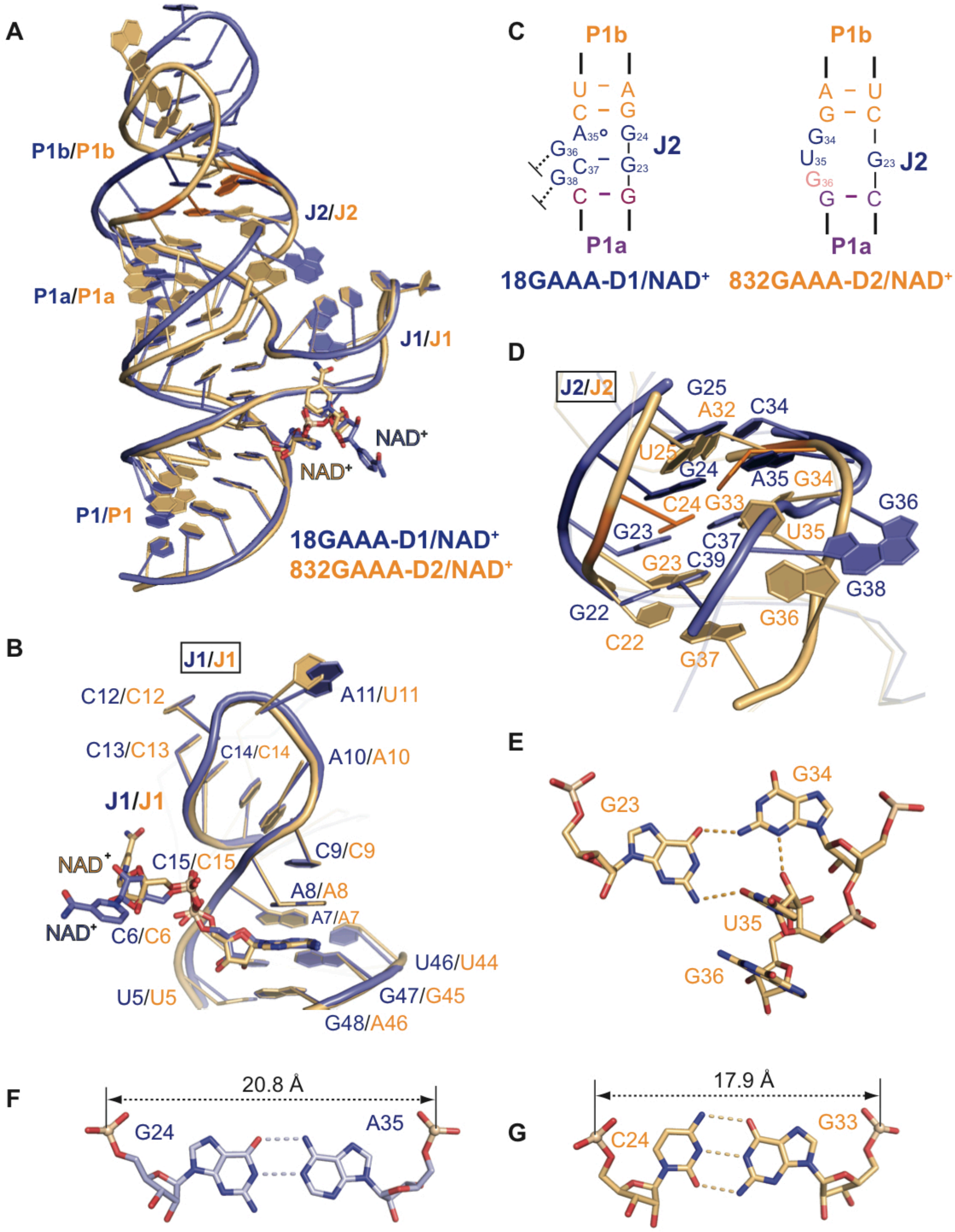

Supplementary Figure S11. Superposition of NAD+-bound 18GAAA-D1 domain 1 and NAD+-bound 832GAAA-D2 domain 2 RNA structures. (**A**) Superposition of domain 1 and domain 2 RNA tertiary structures. (**B**) Close-up view of superposition of junctions J1 and the ligand binding pockets. The NAD+-bound pockets adopt similar conformations, including the ADP moieties of the ligands. The NMN moieties of NAD+ ligands that do not interact with their pockets are in different orientation. (**C**) Juxtaposition of the sequences (and secondary structures) of junctions J2 of domain 1 and domain 2 RNAs. (**D**) Superposition of junctions J2 of domain 1 and domain 2 RNAs. G36 and G38 are directed outwards and form long-distance base-pairs with J1 in domain 1, while the corresponding nucleotides G34 and G36 in domain 2 are directed inwards. (**E**) Close-up view

of the nucleobase interactions in junction J2. (**F**) The non-canonical base pair A35•G24 in junction J2 of domain 1. (**G**) The Watson-Crick base pair G33-C24 in domain 2 takes the place of domain 1 A35•G24 in junction J2, reducing the base pair width significantly.

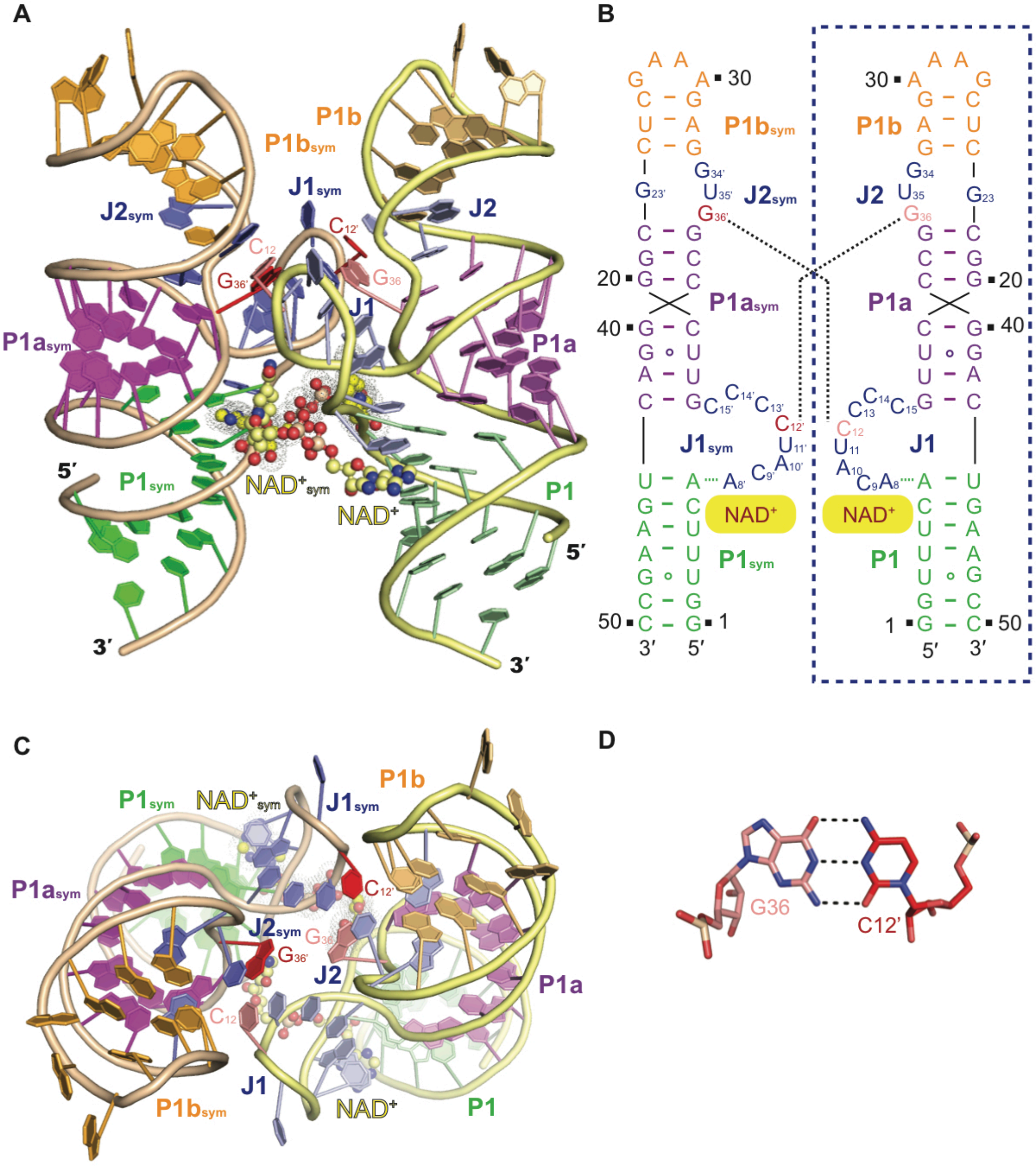

Supplementary Figure S12. Homodimer formation of NAD$^+$ bound 832GAAA-D2 domain 2 RNA. (**A**) Overall tertiary structure of the homodimer of the 832GAAA-D2 RNA observed in the crystal. The interactions at the interface involve both J1 and J2. (**B**) Secondary structure scheme with tertiary interactions indicated as observed in the crystal. (**C**) Same as (A) but from different viewing angle. The interface of the dimer was formed symmetrically by stacking interaction and hydrogen bonding interaction. (**D**) G36 of J2 from one molecule forms a Watson-Crick base pair with C12' of J1 from the symmetry-related molecule.

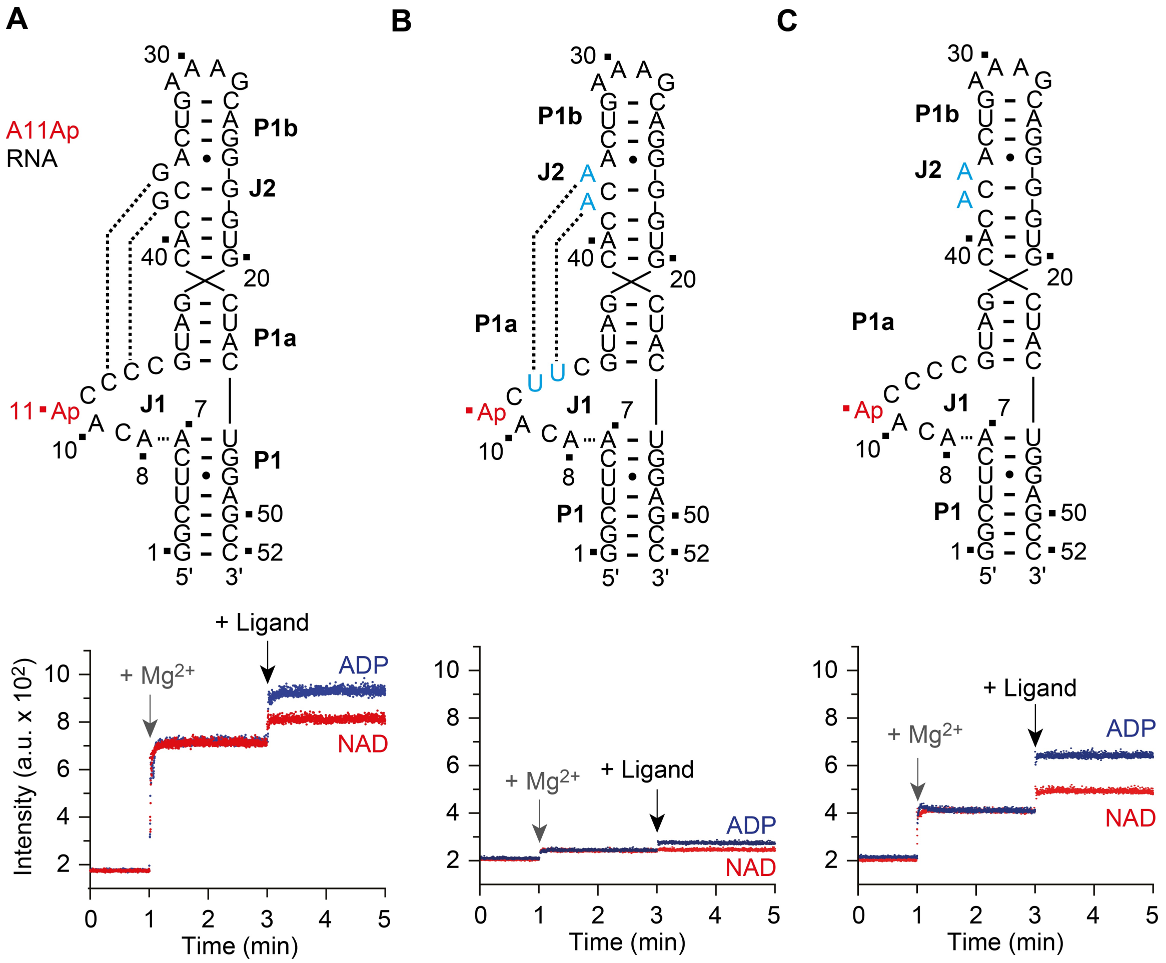

Supplementary Figure S13. Fluorescence assays of mutant domain 1 RNAs. (**A**) Sequence and fluorescence response of the wildtype RNA upon Mg^{2+} and ligand addition (reference system). (**B**) Exchange of CG base pairs by AU base pairs in the long distance interaction (C13U/C14U/G36A/G38A mutant) results in loss of binding. (**C**) Disruption of CG base pairs in the long distance interaction but retaining the C_4 stack in J1 (G36A/G38A mutant) retains binding activity to some extent. For conditions see Figures 5 and 6.

Supplementary Tables S1-S3

Supplementary Table S1. The sequences of the RNA used in structure determination.

Sample	Source	Sequence
17delU1A (Domain1)	Acidobacterium capsulatum ATCC 5119 (NC_012483.1)	5'GGCGCAACAUCCCCGCCGGUU GGGCGCAUUGCACUCCGCGCAG UGAACCGGCUGCGCC3'
832GAAA (Domain2)	Acidobacteriaceae bacterium KBS 83 (KB906733.1)	5'GGUUUCAACAUCCCCGUUCGG CGCUCGAAAGAGGUGGCCGGAC UGAAGCC3'
18GAAA (Domain1)	Rfam family: RF03013	5'GGCUUCAACAACCCCGUAGGU GGGGACGAAAGUCAGCGCACCU ACUGGAGCC3'

Supplementary Table S2. Crystallographic statistics of NAD$^+$ riboswitch (1)

	17delU1A Bound to NAD$^+$	18GAAA Bound to NAD$^+$	832GAAA Bound to NAD$^+$
**Data collection**			
Space group	$P\,6_1\,2\,2$	$I\,2\,2\,2$	$P\,3_2\,2\,1$
Cell dimensions			
a, b, c (Å)	68.6, 68.6, 333.8	55.4, 57.4, 196.4	76.0, 76.0, 50.5
α, β, γ (°)	90, 90, 120	90, 90, 90	90, 90, 120
Wavelength (Å)	0.979	0.979	0.979
Resolution (Å)	50.0-2.8 (2.9-2.8)*	50.0-2.4 (2.44-2.4)	50.0-2.1 (2.18-2.1)
R_{pim}	0.02 (0.15)	0.02 (0.43)	0.02 (0.28)
$I / \sigma I$	33.0 (2.2)	34.0 (1.5)	43.7 (1.6)
Completeness (%)	99.1 (98.4)	98.2 (96.8)	99.8 (99.1)
Redundancy	21.4 (19.8)	12.8 (12.1)	18.9 (16.2)
CC(1/2)	1.0 (0.96)	1.0 (0.93)	0.98 (0.88)
**Refinement**			
Resolution (Å)	27.8-2.8 (2.9-2.8)	25.0-2.4 (2.5-2.4)	30.4-2.1 (2.18-2.1)
No. reflections	12306	12594	10087
R_{work} / R_{free} (%)	25.0/30.8	21.7/23.6	22.4/25.1
No. of atoms			
Protein	746		
RNA	1242	1119	1068
Mg^{2+}	8	11	3
Ligand	44	44	44
Water	41	43	27
Mn^{2+}			
B-factors (Å^2)			
Protein	104.1		
RNA	60.7	84.6	69.1
Mg^{2+}	57.0	85.6	58.1
Ligand	78.6	101.3	74.7
Water	56.8	77.5	52.6
Mn^{2+}			
R.M.S. deviations			
Bond length (Å)	0.003	0.004	0.005
Bond angles (°)	0.76	1.10	1.18
Ramachandran values			
Most favored (%)	80.00		
Additional allowed (%)	14.4		
Outliers (%)	5.6		

*Values in parentheses are for highest-resolution shell.

Supplementary Table S2. Crystallographic statistics of NAD$^+$ riboswitch (2)

	18GAAA Bound to NAD+(Mn)	832GAAA Bound to NAD+(Mn)	18GAAA Bound to ADP	18GAAA Bound to ATP
**Data collection**				
Space group	*I* 2 2 2	*P* 3_2 2 1	*I* 2 2 2	*I* 2 2 2
Cell dimensions				
a, b, c (Å)	55.8, 57.3, 196.6	75.9, 75.9, 50.3	55.3, 57.4, 196.8	56.5, 57.7, 196.8
α, β, γ (°)	90, 90, 90	90, 90, 120	90, 90, 90	90, 90, 90
Wavelength (Å)	1.23980	1.23980	0.97930	0.97930
Resolution (Å)	30.0-2.60 (2.69-2.60)*	30.0-2.50 (2.54-2.50)	50.0-2.64 (2.73-2.64)	50.0-2.40 (2.49-2.40)
R_{pim}	0.04 (0.41)	0.05 (0.47)	0.03 (0.29)	0.04 (0.42)
$I / \sigma I$	16.0 (1.3)	12.2 (1.0)	25.8 (1.3)	19.6 (1.0)
Completeness (%)	99.2 (99.1)	99.3 (96.0)	97.8 (98.5)	98.5 (98.1)
Redundancy	13.0 (13.7)	19.2 (16.2)	12.0 (9.9)	12.1 (9.7)
CC(1/2)	0.98 (0.81)	0.99 (0.78)	1.00 (0.90)	0.99 (0.78)
**Refinement**				
Resolution (Å)	28.7-2.60 (2.69-2.60)	27.5-2.49 (2.58-2.49)	26.6-2.63 (2.73-2.63)	28.3-2.80 (2.90-2.80)
No. reflections	10067	6058	9493	8167
R_{work} / R_{free} (%)	21.3/24.4	22.1/27.1	22.2/24.3	22.0/24.0
No. of atoms				
Protein				
RNA	1123	1068	1123	1123
Mg^{2+}	11	7	8	8
Ligand	44	44	27	31
Water	108	36	40	42
Mn^{2+}	16	7		
B-factors (Å^2)				
Protein				
RNA	58.0	59.9	79.9	70.2
Mg^{2+}	66.6	65.2	78.9	67.6
Ligand	81.9	58.6	79.2	75.1
Water	57.5	50.4	78.1	67.2
Mn^{2+}	70.5	63.4		
R.M.S. deviations				
Bond length (Å)	0.005	0.005	0.006	0.003
Bond angles (°)	1.2	1.2	1.2	0.8
Ramachandran values				
Most favored (%)				
Additional allowed (%)				
Outliers (%)				

*Values in parentheses are for highest-resolution shell.

Supplementary Table S3. Thermodynamic parameters of the binding activity of NAD^+ riboswitch and mutants determined by isothermal titration calorimetry (ITC).

RNA (18GAAA)	Ligand	△H [kcal/mol]	-T△S [kcal/mol]	△G [kcal/mol]	△△G [kcal/mol]	N[a]	K_d[b] [μM]	c-value	K_d[c] (Mean) [μM]
WT	NAD^+	-12.8 ± 1.6	7.5	-5.3	--	1.0 ± 0.06	137 ± 18.2	0.7	127±9.5
		-12.1 ± 0.5	6.7	-5.3		1.0 ± 0.02	126 ± 5.8	0.8	
		-11.5 ± 0.6	6.2	-5.4		1.0 ± 0.02	118 ± 7.1	0.8	
	ADP	-16.3 ± 2.2	10.8	-5.5	0.2	0.8 ± 0.06	95.1 ± 14.3	1.1	95.3±0.3
		-15.8 ± 1.6	10.3	-5.5		0.9 ± 0.04	95.6 ± 11.0	1.1	
		-16.7 ± 1.3	11.2	-5.5		0.8 ± 0.03	95.3 ± 8.1	1.1	
	ATP	-11.0 ± 1.0	5.4	-5.6	0.2	1.0 ± 0.04	82.7 ± 9.9	1.2	90.2±8.6
		-12.5 ± 0.6	6.9	-5.5		0.9 ± 0.02	88.3 ± 5.6	1.1	
		-13.7 ± 0.7	8.3	-5.5		0.8 ± 0.02	99.6 ± 5.2	1.0	
	NADH	-8.0 ± 6.3	3.2	-4.8	-0.5	1.1 ± 0.5	290 ± 160	0.4	305±46.4
		-10.4 ± 8.6	5.7	-4.7		1.0 ± 0.5	357 ± 163	0.3	
		-7.5 ± 4.0	2.6	-4.9		1.1 ± 0.3	268 ± 102	0.4	
	NMN						N.D.[d]		
C6U/G47A	NAD^+						N.D.		
C6G/G47C	NAD^+						N.D.		
G23A/C37U	NAD^+						N.D.		
C13U/C14U /G36A/G38A	NAD^+						N.D.		
C14G/G38C	NAD^+						N.D.		
C13G/G36C	NAD^+						N.D.		
A8U	NAD^+						N.D.		
A8G	NAD^+						N.D.		
A10G	NAD^+						N.D.		
A10C	NAD^+						N.D.		
A11U	NAD^+	-10.5 ± 2.3	5.1	-5.4	0.1	0.9 ± 0.1	111 ± 24.6	0.9	
A35C	NAD^+						N.D.		
A36C	NAD^+						N.D.		
A38C	NAD^+						N.D.		
G36C/G38C	NAD^+						N.D.		

[a] N, the stoichiometric ratio of ligand to RNA.
[b] The binding disassociation parameter fitted for each independent titration.
[c] Reported error is the standard deviation of three independent experiments.
[d] N.D. refers to no detectable interaction under the experiment conditions.
