# Supplementary material for: Structural distinctions between NAD+ riboswitch domains 1 and 2 determine differential folding and ligand binding
Source: Nucleic Acids Res. 2020 Nov 10;48(21):12394–406. doi: 10.1093/nar/gkaa1029 (PMC7708056; doi:10.1093/nar/gkaa1029)
Supplement: gkaa1029_Supplemental_Files [file gkaa1029_supplemental_files.zip › Structural Data.zip!/D_9100043799_17delU1A_NAD_val-report-full_P1.pdf]

Preliminary Full wwPDB X-ray Structure Validation Report ⓘ

Jun 7, 2020 – 10:11 PM EDT

This is a Preliminary Full wwPDB X-ray Structure Validation Report.

This report is produced by the standalone wwPDB validation server.
The structure in question has not been deposited to the wwPDB.
This report should not be submitted to journals.

We welcome your comments at *validation@mail.wwpdb.org*
A user guide is available at
https://www.wwpdb.org/validation/2017/XrayValidationReportHelp
with specific help available everywhere you see the ⓘ symbol.

PRELIMINARY VALIDATION REPORT

The following versions of software and data (see references ⓘ) were used in the production of this report:

MolProbity	:	4.02b-467
Mogul	:	1.8.5 (274361), CSD as541be (2020)
Xtriage (Phenix)	:	1.13
EDS	:	2.12
buster-report	:	1.1.7 (2018)
Percentile statistics	:	20191225.v01 (using entries in the PDB archive December 25th 2019)
Refmac	:	5.8.0158
CCP4	:	7.0.044 (Gargrove)
Ideal geometry (proteins)	:	Engh & Huber (2001)
Ideal geometry (DNA, RNA)	:	Parkinson et al. (1996)
Validation Pipeline (wwPDB-VP)	:	2.12

1 Overall quality at a glance (i)

The following experimental techniques were used to determine the structure:
X-RAY DIFFRACTION

The reported resolution of this entry is 2.80 Å.

Percentile scores (ranging between 0-100) for global validation metrics of the entry are shown in the following graphic. The table shows the number of entries on which the scores are based.

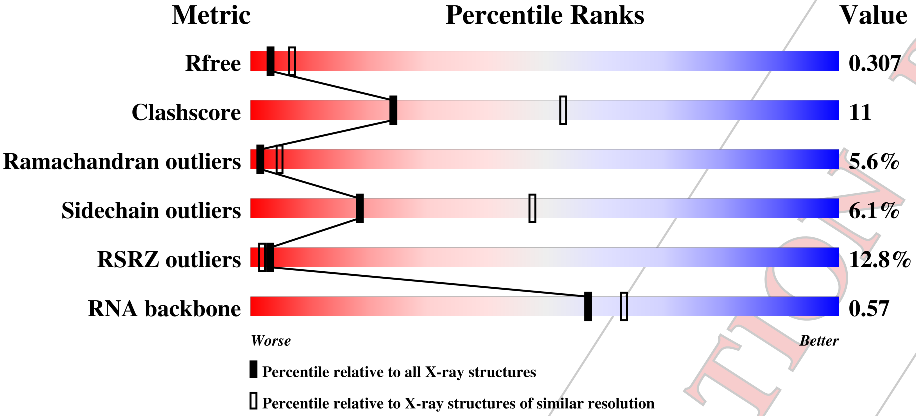

Metric	Whole archive (#Entries)	Similar resolution (#Entries, resolution range(Å))
R_{free}	130704	3140 (2.80-2.80)
Clashscore	141614	3569 (2.80-2.80)
Ramachandran outliers	138981	3498 (2.80-2.80)
Sidechain outliers	138945	3500 (2.80-2.80)
RSRZ outliers	127900	3078 (2.80-2.80)
RNA backbone	3102	1227 (3.10-2.50)

The table below summarises the geometric issues observed across the polymeric chains and their fit to the electron density. The red, orange, yellow and green segments on the lower bar indicate the fraction of residues that contain outliers for >=3, 2, 1 and 0 types of geometric quality criteria respectively. A grey segment represents the fraction of residues that are not modelled. The numeric value for each fraction is indicated below the corresponding segment, with a dot representing fractions <=5% The upper red bar (where present) indicates the fraction of residues that have poor fit to the electron density. The numeric value is given above the bar.

Mol	Chain	Length	Quality of chain
1	A	58	2%<br>67% 22% 10%
2	C	92	20%<br>52% 40% 8%

PRELIMINARY VALIDATION REPORT

2 Entry composition (i)

There are 5 unique types of molecules in this entry. The entry contains 2081 atoms, of which 0 are hydrogens and 0 are deuteriums.

In the tables below, the ZeroOcc column contains the number of atoms modelled with zero occupancy, the AltConf column contains the number of residues with at least one atom in alternate conformation and the Trace column contains the number of residues modelled with at most 2 atoms.

- Molecule 1 is a RNA chain called RNA (58-MER).

Mol	Chain	Residues	Atoms					ZeroOcc	AltConf	Trace
			Total	C	N	O	P			
1	A	58	1242	549	220	413	60	0	0	0

- Molecule 2 is a protein.

Mol	Chain	Residues	Atoms					ZeroOcc	AltConf	Trace
			Total	C	N	O	S			
2	C	92	746	477	132	134	3	0	0	0

- Molecule 3 is MAGNESIUM ION (three-letter code: MG) (formula: Mg).

Mol	Chain	Residues	Atoms		ZeroOcc	AltConf
			Total	Mg		
3	D	1	1	1	1	0
3	D	1	1	1	1	0
3	D	1	1	1	1	0
3	D	1	1	1	1	0
3	D	1	1	1	1	0
3	D	1	1	1	1	0
3	D	1	1	1	0	0
3	D	1	1	1	0	0

- Molecule 4 is NICOTINAMIDE-ADENINE-DINUCLEOTIDE (three-letter code: NAD) (formula: $C_{21}H_{27}N_7O_{14}P_2$).

PRELIMINARY VALIDATION REPORT

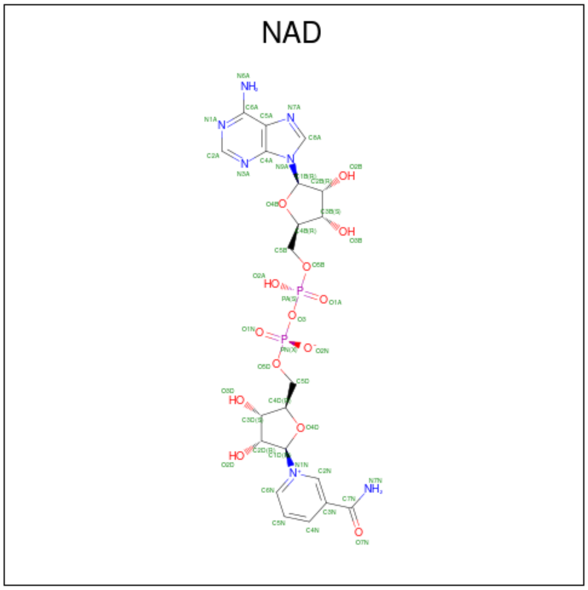

Mol	Chain	Residues	Atoms					ZeroOcc	AltConf
			Total	C	N	O	P		
4	B	1	44	21	7	14	2	0	0

- Molecule 5 is water.

Mol	Chain	Residues	Atoms		ZeroOcc	AltConf
			Total	O		
5	E	41	41	41	0	0

PRELIMINARY VALIDATION REPORT

3 Residue-property plots (i)

These plots are drawn for all protein, RNA and DNA chains in the entry. The first graphic for a chain summarises the proportions of the various outlier classes displayed in the second graphic. The second graphic shows the sequence view annotated by issues in geometry and electron density. Residues are color-coded according to the number of geometric quality criteria for which they contain at least one outlier: green = 0, yellow = 1, orange = 2 and red = 3 or more. A red dot above a residue indicates a poor fit to the electron density (RSRZ > 2). Stretches of 2 or more consecutive residues without any outlier are shown as a green connector. Residues present in the sample, but not in the model, are shown in grey.

- Molecule 1: RNA (58-MER)

Chain A:

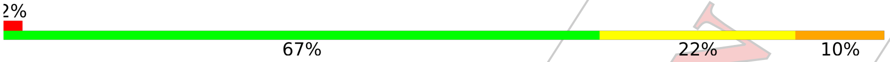

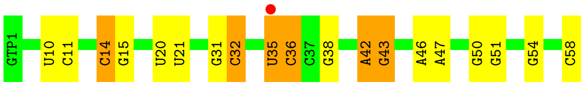

- Molecule 2:

Chain C:

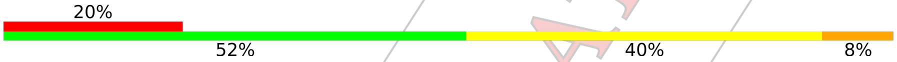

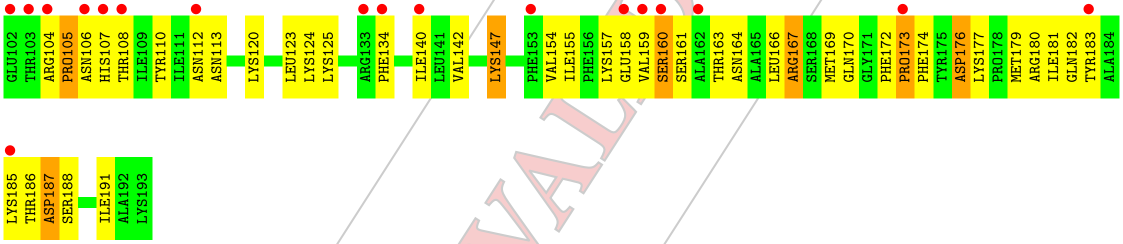

PRELIMINARY VALIDATION REPORT

4 Data and refinement statistics (i)

Property	Value	Source
Space group	P 61 2 2	Depositor
Cell constants<br>a, b, c, α, β, γ	68.59Å 68.59Å 333.85Å<br>90.00° 90.00° 120.00°	Depositor
Resolution (Å)	27.82 – 2.80<br>27.82 – 2.80	Depositor<br>EDS
% Data completeness<br>(in resolution range)	99.1 (27.82-2.80)<br>99.1 (27.82-2.80)	Depositor<br>EDS
R_{merge}	0.08	Depositor
R_{sym}	(Not available)	Depositor
$< I/\sigma(I) >$ [1]	2.44 (at 2.80Å)	Xtriage
Refinement program	PHENIX 1.14_3260	Depositor
R, R_{free}	0.250 , 0.308<br>0.250 , 0.307	Depositor<br>DCC
R_{free} test set	603 reflections (4.90%)	wwPDB-VP
Wilson B-factor (Å^2)	70.0	Xtriage
Anisotropy	0.608	Xtriage
Bulk solvent k_{sol}(e/Å^3), B_{sol}(Å^2)	0.29 , 42.2	EDS
L-test for twinning[2]	$< \|L\| > = 0.48$, $< L^2 > = 0.32$	Xtriage
Estimated twinning fraction	No twinning to report.	Xtriage
F_o,F_c correlation	0.93	EDS
Total number of atoms	2081	wwPDB-VP
Average B, all atoms (Å^2)	76.0	wwPDB-VP

Xtriage's analysis on translational NCS is as follows: *The largest off-origin peak in the Patterson function is 4.87% of the height of the origin peak. No significant pseudotranslation is detected.*

[1] Intensities estimated from amplitudes.

[2] Theoretical values of $< |L| >$, $< L^2 >$ for acentric reflections are 0.5, 0.333 respectively for untwinned datasets, and 0.375, 0.2 for perfectly twinned datasets.

PRELIMINARY VALIDATION REPORT

5 Model quality (i)

5.1 Standard geometry (i)

Bond lengths and bond angles in the following residue types are not validated in this section: GTP, MG, NAD

The Z score for a bond length (or angle) is the number of standard deviations the observed value is removed from the expected value. A bond length (or angle) with $|Z| > 5$ is considered an outlier worth inspection. RMSZ is the root-mean-square of all Z scores of the bond lengths (or angles).

Mol	Chain	Bond lengths		Bond angles	
		RMSZ	#$|Z|$ >5	RMSZ	#$|Z|$ >5
1	A	0.23	0/1349	0.74	0/2100
2	C	0.28	0/759	0.48	0/1017
All	All	0.25	0/2108	0.66	0/3117

Chiral center outliers are detected by calculating the chiral volume of a chiral center and verifying if the center is modelled as a planar moiety or with the opposite hand.A planarity outlier is detected by checking planarity of atoms in a peptide group, atoms in a mainchain group or atoms of a sidechain that are expected to be planar.

Mol	Chain	#Chirality outliers	#Planarity outliers
2	C	0	1

There are no bond length outliers.

There are no bond angle outliers.

There are no chirality outliers.

All (1) planarity outliers are listed below:

Mol	Chain	Res	Type	Group
2	C	104	ARG	Peptide

5.2 Too-close contacts (i)

In the following table, the Non-H and H(model) columns list the number of non-hydrogen atoms and hydrogen atoms in the chain respectively. The H(added) column lists the number of hydrogen atoms added and optimized by MolProbity. The Clashes column lists the number of clashes within the asymmetric unit, whereas Symm-Clashes lists symmetry related clashes.

PRELIMINARY VALIDATION REPORT

Mol	Chain	Non-H	H(model)	H(added)	Clashes	Symm-Clashes
1	A	1242	0	632	12	0
2	C	746	0	766	30	0
3	D	8	0	0	0	0
4	B	44	0	26	1	0
5	E	41	0	0	1	0
All	All	2081	0	1424	38	0

The all-atom clashscore is defined as the number of clashes found per 1000 atoms (including hydrogen atoms). The all-atom clashscore for this structure is 11.

All (38) close contacts within the same asymmetric unit are listed below, sorted by their clash magnitude.

Atom-1	Atom-2	Interatomic distance (Å)	Clash overlap (Å)
2:C:164:ASN:HA	2:C:167:ARG:HG3	1.63	0.79
2:C:157:LYS:HD3	2:C:158:GLU:HG2	1.74	0.68
2:C:158:GLU:N	2:C:161:SER:OG	2.24	0.67
1:A:31:G:N7	2:C:112:ASN:ND2	2.38	0.65
2:C:105:PRO:HD2	2:C:183:TYR:HB2	1.82	0.60
2:C:108:THR:OG1	2:C:154:VAL:O	2.22	0.57
2:C:107:HIS:CE1	2:C:188:SER:H	2.22	0.57
1:A:35:U:H2'	1:A:36:C:H5"	1.87	0.55
1:A:50:G:N7	5:E:9:HOH:O	2.33	0.55
2:C:147:LYS:HD2	2:C:147:LYS:H	1.72	0.54
2:C:159:VAL:HG13	2:C:160:SER:H	1.73	0.54
2:C:107:HIS:CE1	2:C:187:ASP:HA	2.45	0.52
2:C:108:THR:OG1	2:C:155:ILE:HA	2.11	0.51
2:C:187:ASP:HB2	2:C:191:ILE:HD11	1.93	0.50
1:A:32:C:N4	2:C:182:GLN:HG2	2.26	0.50
2:C:160:SER:HA	2:C:163:THR:HG23	1.93	0.49
2:C:166:LEU:HD13	2:C:181:ILE:HB	1.94	0.48
2:C:172:PHE:O	2:C:174:PHE:N	2.46	0.48
2:C:186:THR:OG1	2:C:187:ASP:N	2.48	0.47
1:A:14:C:H5"	1:A:15:G:OP2	2.15	0.47
2:C:140:ILE:HG13	2:C:154:VAL:HG22	1.97	0.46
2:C:169:MET:HB2	2:C:181:ILE:HD11	1.96	0.46
1:A:20:U:H2'	1:A:21:U:C6	2.50	0.46
1:A:32:C:H41	2:C:182:GLN:HG2	1.79	0.46
1:A:50:G:H2'	1:A:51:G:O4'	2.15	0.46
2:C:172:PHE:HD1	2:C:173:PRO:HD2	1.82	0.44
2:C:113:ASN:HD21	2:C:177:LYS:HE2	1.82	0.44
2:C:123:LEU:HD23	2:C:142:VAL:HG11	1.99	0.43
2:C:179:MET:O	2:C:180:ARG:HG3	2.18	0.43

Continued on next page...

PRELIMINARY VALIDATION REPORT

Continued from previous page...

Atom-1	Atom-2	Interatomic distance (Å)	Clash overlap (Å)
2:C:159:VAL:HG13	2:C:160:SER:N	2.33	0.43
1:A:42:A:O2'	1:A:43:G:OP1	2.32	0.43
2:C:125:LYS:HE3	2:C:125:LYS:HB2	1.81	0.43
2:C:110:TYR:N	2:C:182:GLN:O	2.52	0.42
1:A:54:G:N3	4:B:1:NAD:H2A	2.34	0.42
1:A:46:A:H2'	1:A:47:A:C8	2.55	0.41
2:C:120:LYS:HE3	2:C:124:LYS:HE3	2.03	0.41
1:A:32:C:N3	2:C:185:LYS:HG3	2.36	0.41
2:C:176:ASP:OD1	2:C:176:ASP:N	2.53	0.40

There are no symmetry-related clashes.

5.3 Torsion angles (i)

5.3.1 Protein backbone (i)

In the following table, the Percentiles column shows the percent Ramachandran outliers of the chain as a percentile score with respect to all X-ray entries followed by that with respect to entries of similar resolution.

The Analysed column shows the number of residues for which the backbone conformation was analysed, and the total number of residues.

Mol	Chain	Analysed	Favoured	Allowed	Outliers	Percentiles
2	C	90/92 (98%)	72 (80%)	13 (14%)	5 (6%)	2 5

All (5) Ramachandran outliers are listed below:

Mol	Chain	Res	Type
2	C	105	PRO
2	C	170	GLN
2	C	167	ARG
2	C	106	ASN
2	C	173	PRO

5.3.2 Protein sidechains (i)

In the following table, the Percentiles column shows the percent sidechain outliers of the chain as a percentile score with respect to all X-ray entries followed by that with respect to entries of similar resolution.

PRELIMINARY VALIDATION REPORT

The Analysed column shows the number of residues for which the sidechain conformation was analysed, and the total number of residues.

Mol	Chain	Analysed	Rotameric	Outliers	Percentiles	
2	C	82/83 (99%)	77 (94%)	5 (6%)	18	48

All (5) residues with a non-rotameric sidechain are listed below:

Mol	Chain	Res	Type
2	C	134	PHE
2	C	147	LYS
2	C	160	SER
2	C	176	ASP
2	C	187	ASP

Some sidechains can be flipped to improve hydrogen bonding and reduce clashes. There are no such sidechains identified.

5.3.3 RNA (i)

Mol	Chain	Analysed	Backbone Outliers	Pucker Outliers
1	A	56/58 (96%)	9 (16%)	1 (1%)

All (9) RNA backbone outliers are listed below:

Mol	Chain	Res	Type
1	A	10	U
1	A	11	C
1	A	14	C
1	A	32	C
1	A	35	U
1	A	36	C
1	A	38	G
1	A	43	G
1	A	58	C

All (1) RNA pucker outliers are listed below:

Mol	Chain	Res	Type
1	A	42	A

PRELIMINARY VALIDATION REPORT

5.4 Non-standard residues in protein, DNA, RNA chains (i)

There are no non-standard protein/DNA/RNA residues in this entry.

5.5 Carbohydrates (i)

There are no carbohydrates in this entry.

5.6 Ligand geometry (i)

Of 9 ligands modelled in this entry, 8 are monoatomic - leaving 1 for Mogul analysis.

In the following table, the Counts columns list the number of bonds (or angles) for which Mogul statistics could be retrieved, the number of bonds (or angles) that are observed in the model and the number of bonds (or angles) that are defined in the Chemical Component Dictionary. The Link column lists molecule types, if any, to which the group is linked. The Z score for a bond length (or angle) is the number of standard deviations the observed value is removed from the expected value. A bond length (or angle) with $|Z| > 2$ is considered an outlier worth inspection. RMSZ is the root-mean-square of all Z scores of the bond lengths (or angles).

Mol	Type	Chain	Res	Link	Bond lengths			Bond angles		
					Counts	RMSZ	$\#\|Z\| > 2$	Counts	RMSZ	$\#\|Z\| > 2$
4	NAD	B	1	3	42,48,48	0.81	1 (2%)	50,73,73	1.16	5 (10%)

In the following table, the Chirals column lists the number of chiral outliers, the number of chiral centers analysed, the number of these observed in the model and the number defined in the Chemical Component Dictionary. Similar counts are reported in the Torsion and Rings columns. '-' means no outliers of that kind were identified.

Mol	Type	Chain	Res	Link	Chirals	Torsions	Rings
4	NAD	B	1	3	-	11/26/62/62	0/5/5/5

All (1) bond length outliers are listed below:

Mol	Chain	Res	Type	Atoms	Z	Observed(Å)	Ideal(Å)
4	B	1	NAD	C5A-C4A	2.46	1.47	1.40

All (5) bond angle outliers are listed below:

Mol	Chain	Res	Type	Atoms	Z	Observed(^{o})	Ideal(^{o})
4	B	1	NAD	N3A-C2A-N1A	-3.06	123.89	128.68
4	B	1	NAD	C3B-C2B-C1B	2.91	105.36	100.98
4	B	1	NAD	C4A-C5A-N7A	-2.91	106.37	109.40

Continued on next page...

PRELIMINARY VALIDATION REPORT

Continued from previous page...

Mol	Chain	Res	Type	Atoms	Z	Observed(^{o})	Ideal(^{o})
4	B	1	NAD	PN-O3-PA	-2.84	123.09	132.83
4	B	1	NAD	C3D-C2D-C1D	2.79	105.18	100.98

There are no chirality outliers.

All (11) torsion outliers are listed below:

Mol	Chain	Res	Type	Atoms
4	B	1	NAD	C5B-O5B-PA-O2A
4	B	1	NAD	C5D-O5D-PN-O1N
4	B	1	NAD	O4D-C4D-C5D-O5D
4	B	1	NAD	O4D-C1D-N1N-C2N
4	B	1	NAD	O4D-C1D-N1N-C6N
4	B	1	NAD	C4N-C3N-C7N-O7N
4	B	1	NAD	C2N-C3N-C7N-O7N
4	B	1	NAD	C2N-C3N-C7N-N7N
4	B	1	NAD	C4N-C3N-C7N-N7N
4	B	1	NAD	C5B-O5B-PA-O3
4	B	1	NAD	C5B-O5B-PA-O1A

There are no ring outliers.

1 monomer is involved in 1 short contact:

Mol	Chain	Res	Type	Clashes	Symm-Clashes
4	B	1	NAD	1	0

The following is a two-dimensional graphical depiction of Mogul quality analysis of bond lengths, bond angles, torsion angles, and ring geometry for all instances of the Ligand of Interest. In addition, ligands with molecular weight > 250 and outliers as shown on the validation Tables will also be included. For torsion angles, if less then 5% of the Mogul distribution of torsion angles is within 10 degrees of the torsion angle in question, then that torsion angle is considered an outlier. Any bond that is central to one or more torsion angles identified as an outlier by Mogul will be highlighted in the graph. For rings, the root-mean-square deviation (RMSD) between the ring in question and similar rings identified by Mogul is calculated over all ring torsion angles. If the average RMSD is greater than 60 degrees and the minimal RMSD between the ring in question and any Mogul-identified rings is also greater than 60 degrees, then that ring is considered an outlier. The outliers are highlighted in purple. The color gray indicates Mogul did not find sufficient equivalents in the CSD to analyse the geometry.

PRELIMINARY VALIDATION REPORT

Ligand NAD B 1

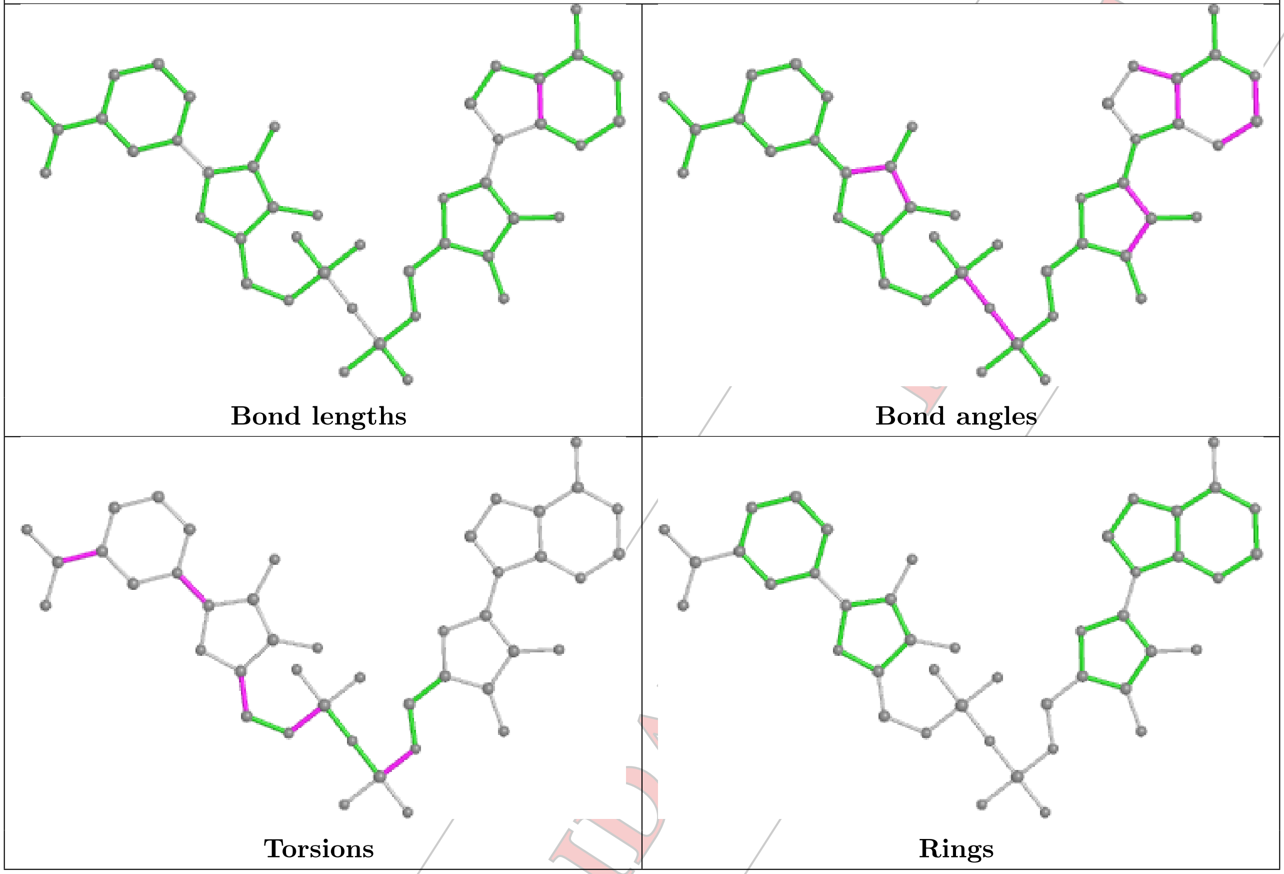

5.7 Other polymers (i)

There are no such residues in this entry.

5.8 Polymer linkage issues (i)

The following chains have linkage breaks:

Mol	Chain	Number of breaks
1	A	1

All chain breaks are listed below:

Model	Chain	Residue-1	Atom-1	Residue-2	Atom-2	Distance (Å)
1	A	1:GTP	O3’	2:G	P	3.11

PRELIMINARY

6 Fit of model and data (i)

6.1 Protein, DNA and RNA chains (i)

In the following table, the column labelled '#RSRZ> 2' contains the number (and percentage) of RSRZ outliers, followed by percent RSRZ outliers for the chain as percentile scores relative to all X-ray entries and entries of similar resolution. The OWAB column contains the minimum, median, 95th percentile and maximum values of the occupancy-weighted average B-factor per residue. The column labelled 'Q< 0.9' lists the number of (and percentage) of residues with an average occupancy less than 0.9.

Mol	Chain	Analysed	<RSRZ>	#RSRZ>2			OWAB(Å^2)	Q<0.9
1	A	57/58 (98%)	-0.57	1 (1%)	68	61	47, 57, 93, 104	0
2	C	92/92 (100%)	1.05	18 (19%)	1	0	59, 107, 130, 137	0
All	All	149/150 (99%)	0.43	19 (12%)	3	2	47, 89, 129, 137	0

All (19) RSRZ outliers are listed below:

Mol	Chain	Res	Type	RSRZ
2	C	159	VAL	6.0
2	C	133	ARG	4.6
2	C	173	PRO	3.7
2	C	183	TYR	3.2
2	C	158	GLU	3.1
2	C	160	SER	3.0
2	C	112	ASN	2.9
2	C	103	THR	2.9
2	C	162	ALA	2.8
2	C	106	ASN	2.6
2	C	104	ARG	2.6
2	C	134	PHE	2.6
1	A	35	U	2.5
2	C	140	ILE	2.4
2	C	102	GLU	2.4
2	C	108	THR	2.4
2	C	153	PHE	2.3
2	C	107	HIS	2.2
2	C	185	LYS	2.1

6.2 Non-standard residues in protein, DNA, RNA chains (i)

There are no non-standard protein/DNA/RNA residues in this entry.

PRELIMINARY VALIDATION REPORT

6.3 Carbohydrates (i)

There are no carbohydrates in this entry.

6.4 Ligands (i)

In the following table, the Atoms column lists the number of modelled atoms in the group and the number defined in the chemical component dictionary. The B-factors column lists the minimum, median, 95th percentile and maximum values of B factors of atoms in the group. The column labelled 'Q< 0.9' lists the number of atoms with occupancy less than 0.9.

Mol	Type	Chain	Res	Atoms	RSCC	RSR	B-factors(Å^2)	Q<0.9
3	MG	D	1	1/?	-	-	50,50,50,50	1
3	MG	D	8	1/?	0.70	0.28	76,76,76,76	0
4	NAD	B	1	44/?	0.92	0.25	51,65,108,110	0
3	MG	D	6	1/?	-	-	58,58,58,58	1
3	MG	D	2	1/?	-	-	50,50,50,50	1
3	MG	D	7	1/?	0.98	0.05	58,58,58,58	0
3	MG	D	5	1/?	-	-	60,60,60,60	1
3	MG	D	4	1/?	-	-	49,49,49,49	1
3	MG	D	3	1/?	-	-	51,51,51,51	1

The following is a graphical depiction of the model fit to experimental electron density of all instances of the Ligand of Interest. In addition, ligands with molecular weight > 250 and outliers as shown on the geometry validation Tables will also be included. Each fit is shown from different orientation to approximate a three-dimensional view.

PRELIMINARY VALIDATION REPORT

Electron density around NAD B 1:

2mF$_o$-DF$_c$ (at 0.7 rmsd) in gray
mF$_o$-DF$_c$ (at 3 rmsd) in purple (negative)
and green (positive)

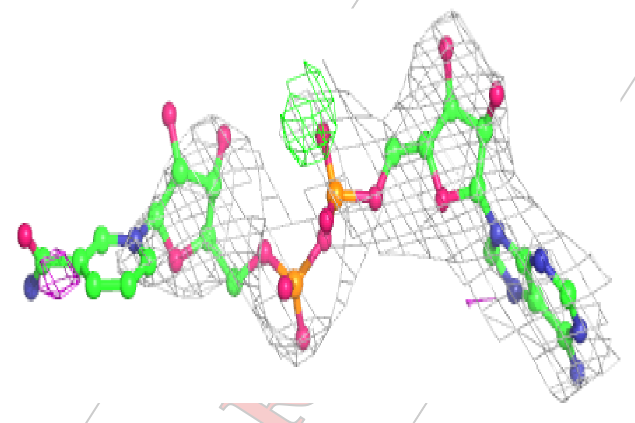

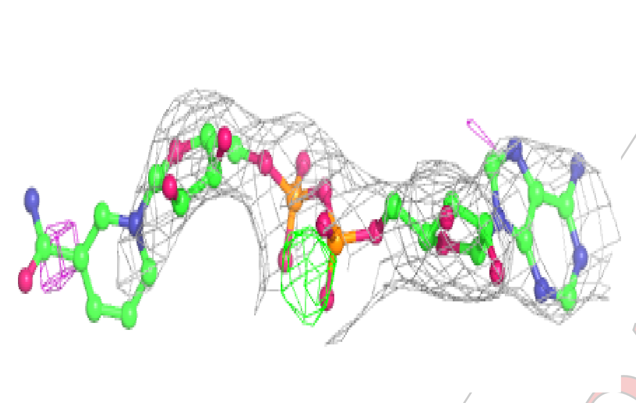

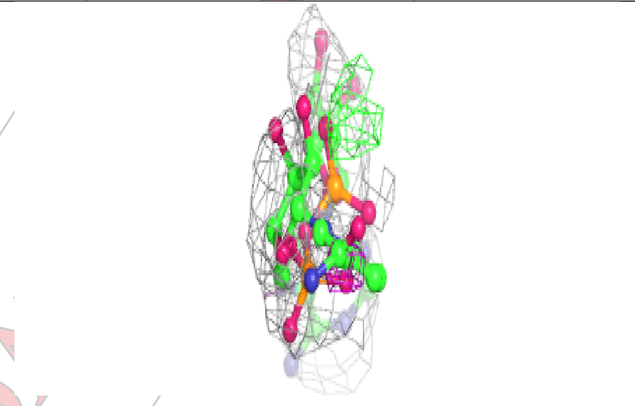

6.5 Other polymers ⓘ

There are no such residues in this entry.

PRELIMINARY VALIDATION
